# Kinetic Modeling of pH-Dependent Oxidation of Dopamine by Iron and Its Relevance to Parkinson's Disease

**DOI:** 10.3389/fnins.2018.00859

**Published:** 2018-11-26

**Authors:** Yingying Sun, A. Ninh Pham, Dominic J. Hare, T. David Waite

**Affiliations:** ^1^Water Research Centre, School of Civil and Environmental Engineering, The University of New South Wales, Sydney, NSW, Australia; ^2^Atomic Pathology Laboratory, Melbourne Dementia Research Centre at the Florey Institute of Neuroscience and Mental Health and The University of Melbourne, Parkville, VIC, Australia; ^3^Department of Clinical Pathology, The University of Melbourne, Parkville, VIC, Australia

**Keywords:** pH, iron, dopamine, aminochrome, oxidative stress, Parkinson's disease

## Abstract

Parkinson's disease is the second most common neurodegenerative disease. While age is the most significant risk factor, the exact cause of this disease and the most effective approaches to mitigation remain unclear. It has long been proposed that dopamine may play a role in the pathology of Parkinson's disease in view of its ability to generate both protein-modifying quinones such as aminochrome and reactive oxygen species, especially in the presence of pathological iron accumulation in the primary site of neuron loss. Given the clinically measured acidosis of *post-mortem* Parkinson's disease brain tissue, the interaction between dopamine and iron was investigated over a pH range of 7.4 to 6.5 with emphasis on the accumulation of toxic quinones and generation of reactive oxygen species. Our results show that the presence of iron accelerates the formation of aminochrome with ferrous iron (Fe[II]) being more efficient in this regard than ferric iron (Fe[III]). Our results further suggest that a reduced aminochrome rearrangement rate coupled with an enhanced turnover rate of Fe[II] as a result of brain tissue acidosis could result in aminochrome accumulation within cells. Additionally, under these conditions, the enhanced redox cycling of iron in the presence of dopamine aggravates oxidative stress as a result of the production of damaging reactive species, including hydroxyl radicals.

## Introduction

Parkinson's disease is the second most common neurodegenerative disorder after Alzheimer's disease (Guttmacher et al., 2003). Even though the exact cause of Parkinson's disease is still unknown, two main pathological indicators are observed in the *post-mortem* brain; namely (i) loss of dopamine-producing neurons in the substantia nigra pars compacta (SNc; Kordower et al., 2013) and (ii) the widespread deposition of amyloid-like Lewy bodies rich in α-synuclein both in the SNc and other brain regions where overt neurodegeneration is not observed (Surmeier et al., 2017).

Accumulation of iron (Fe) in the SNc is an additional pathological feature common to all forms of Parkinson's disease (Ayton and Lei, 2014) that appears to precede the onset of clinical symptoms (Berg et al., 2015). Unlike synucleinopathy, brain Fe accumulation beyond that observed in normal aging is an early-stage event (He et al., 2015) restricted to the degenerating nigrostriatal pathway in Parkinson's disease (Wang et al., 2016) with this association supporting a potential causative role of Fe in neuron loss. In healthy neurons, Fe uptake is primarily *via* transferrin receptor mediated endocytosis, which releases labile Fe into the cytosol for immediate distribution to various organelles and Fe-storage and regulatory proteins. In dopaminergic neurons a small amount of Fe is bound to neuromelanin, a biopolymer formed during the oxidation of dopamine (DA). The labile iron pool is thought to be transient and consists primarily of both ferrous (Fe[II]) and ferric (Fe[III]) species bound to low molecular weight ligands such as citrate and ATP (Double et al., 2002; Hare et al., 2013). While characterization of the labile Fe pool in a living system, particularly human tissue, remains an analytical challenge (New et al., 2018), it has been argued that an increase in reactive Fe in the labile iron pool may contribute to generation of reactive oxygen species (ROS) in Parkinson's disease at levels above those that can normally be attenuated by endogenous antioxidant mechanisms (Ward et al., 2014). Biochemically, Fe-mediated generation of ROS is primarily *via* traditional Fenton/Haber-Weiss chemistry (Equations 1, 2) where cytosolic iron cycles between Fe[II] and Fe[III] to produce a range of harmful oxidants, including superoxide (O${}_{2}^{•-}$), hydrogen peroxide (H_2_O_2_) and hydroxyl radicals (^•^OH; Graham et al., 1978; Halliwell and Gutteridge, 1984; Segura-Aguilar et al., 2014; Sun et al., 2016):

$$\begin{array}{rcl} \text{Fe}\left[\text{III}\right]+{{\text{O}}_{2}}^{•-} & \to & \text{Fe}\left[\text{II}\right]+{\text{O}}_{2} \end{array}$$

$$\begin{array}{rcl} \text{Fe}\left[\text{II}\right]+{\text{H}}_{2}{\text{O}}_{2} & \to & \text{Fe}\left[\text{III}\right]{+}^{•}\text{OH}+\text{O}{\text{H}}^{-} \end{array}$$

The Fenton/Haber-Weiss reaction is not, however, unique to dopaminergic neurons and normal Fe accumulation with age occurs in other neuroanatomical regions (Acosta-Cabronero et al., 2016) without causing generalized cell loss. Numerous antioxidant mechanisms, including superoxide dismutase 1 (SOD1), reduced glutathione (GSH) and glutathione peroxidase, and vitamin E-mediated scavenging of O${}_{2}^{•-}$, H_2_O_2_ and ^•^OH, attenuate potential ROS-induced neurotoxicity from gradual increase in Fe levels, suggesting that dysfunction of attenuating mechanisms is involved in Fe-mediated dopaminergic cell death in Parkinson's disease (Zecca et al., 2004).

An emerging theory, supported by initial observations of Fe dysregulation dating back nearly 100 years (Lhermitte et al., 1924), suggests that abnormal interactions between Fe and DA represent a biochemical mechanism unique to the microchemical environment of vulnerable neurons (Hare et al., 2014). Healthy dopaminergic neurons have particularly high endogenous oxidative load, owing to their extensive and complex axonal network, large soma and high metabolic output (Blesa et al., 2015). Further, cytosolic DA present in the mid-μM range in these neurons (Mosharov et al., 2009) gives rise to a pathway of Fe-mediated ROS generation independent of Fenton/Haber-Weiss chemistry (Hare and Double, 2016). Dopamine-derived quinones, such the DA *o*-quinone (DAQ) and aminochrome (DAC) can also be produced *via* the Fe-driven generation of the transient precursor DA semiquinone (DA^•−^) intermediary (Hare and Double, 2016; Sun et al., 2018b). It is unclear if endogenous antioxidant enzymes are capable of attenuating damage from DA-quinone production, though one proposed mechanism involves polymerization as the dark pigment, neuromelanin (Zhang et al., 2012).

As a major neurotoxic metabolite of DA, DAC has been used as a preclinical model compound to examine neurotoxicity in view of its apparent ability to cause indiscriminate neuronal damage, including mitochondrial dysfunction (Herrera et al., 2016; Segura-Aguilar and Huenchuguala, 2018), likely *via* lipid peroxidation and disruption of membrane integrity. It has been proposed that DAC can induce aggregation of α-synuclein and eventual Lewy body deposition through formation of a DAC-synuclein adduct (Berman and Hastings, 1999; Conway et al., 2001; Bianco et al., 2002; Norris et al., 2005), and α-synuclein mRNA has a predicted iron-response element in the 5′-untranslated region similar to the Fe-storage protein ferritin (Friedlich et al., 2007), suggesting this hallmark protein of Parkinson's disease also plays a role in Fe-mediated toxicity within vulnerable neurons. Previous studies also reported that the presence of DA quinones may also aggravate oxidative stress as a result of the inactivation in the electron transport chain of mitochondrial complexes I and III and the subsequent leakage of electrons from the respiratory chain (Stokes et al., 1999; Adam-Vizi, 2005; Lin and Beal, 2006; Gautier et al., 2008).

Much of our understanding of Fe-mediated DA oxidation has come from *ex vivo* studies where the chemical environment remains relatively constant, or *in vivo* investigations using cell culture or simple animal models of parkinsonism (Jiang et al., 2013; Panicker et al., 2015; Sampson et al., 2016). While useful and of most relevance, *in vivo* experiments generally provide insight into the overall consequence of the whole process with limited insights regarding the exact pathway, or pathways, of DA transformation. As such, it is unlikely that location of the most toxic intermediates or their relative concentrations will be determined in such studies with resultant restrictions in identifying the most efficacious therapeutic strategies targeting toxic DA metabolites. While previous work has firmly established that aberrant reactions between Fe and DA give rise to increased levels of free radicals and oxidative stress markers (Hermida-Ameijeiras et al., 2004; Jiang et al., 2013), comparatively little attention has been paid to the quantitative study of the DA intermediates that are directly implicated in causing neuronal dysfunction. Mitochondrial complex I inhibition by DA quinones and subsequent mitochondrial dysfunction has been identified as a possible molecular basis of neurodegeneration in Parkinson's disease (Schapira, 1994) and resulting intraneuronal acidosis is likely to decrease cytoplasmic pH (Balut et al., 2008) and influence the kinetics of Fe-mediated DA oxidation *in vivo*. Further, while the frequently observed decrease in pH of *post-mortem* brain tissue in the numerous tissue biobanks may result from *ex vivo* handling (Hare et al., 2012), physiological effects of cardiorespiratory failure, cerebrovascular accident and end-stage neurodegenerative diseases generally result in lower brain tissue pH ranging from 7.0 to ~ 6.0 (Hardy et al., 1985; Yates et al., 1990; Harrison et al., 1995; Monoranu et al., 2009; Genoud et al., 2017). Mitochondrial dysfunction, which is a cardinal feature of neurodegenerative disease (Lin and Beal, 2006), also decreases tissue pH *via* leakage of protons into the cytosol (Brand and Nicholls, 2011). Thus, a reduction in brain tissue pH as a result of disease progression may have a substantial role in accelerating the progression of Fe-mediated DA oxidation in Parkinson's disease.

To demonstrate the relevance of interplay between Fe and DA to Parkinson's disease under acidified conditions mimicking predicted effects of disease-specific *pre-mortem* and non-specific *post-mortem* changes, we examined interactions between DA and Fe *in vitro* at a range of pH values–from pH 7.4, the typical pH of extracellular fluid (ECF) to pH 6.5, which is the reported average pH value of human Parkinson's disease tissue from the Sydney Brain Bank (Genoud et al., 2017) (though the specific location within the brain from which these samples were drawn was not reported). In the majority of previous studies, biochemical assays were undertaken at pH 7.4 as opposed to intraneuronal values that are typically around 6.96 to 7.05. This is a major limitation given that the transformation and fate of a range of biochemical compounds such as DA is strongly dependent upon pH. As Fe-mediated DA oxidation is an aerobic process, we also examined the effects of dissolved O_2_ concentration within aqueous systems with respect to both the kinetics of Fe[II] oxidation and the production of reactive DA intermediates. In order to facilitate accurate and precise determination of reactive and unstable quinone intermediates, these investigations of the influence of pH and oxygen concentration on Fe-mediated DA oxidation have been performed *ex vivo*. These fundamental studies are critical for understanding Fe-DA reaction kinetics and set the stage for future studies where the techniques described are applied to the study of both the formation of neurotoxic DA metabolites in the Parkinson's disease brain and to the new therapies designed to prevent abnormal reactions of Fe within dopaminergic neurons that account for the changing pH of a degenerating dopaminergic neuron.

## Materials and methods

### Chemical and reagents

All analytical grade chemicals were purchased from Sigma-Aldrich (Castle Hill, Australia) or as otherwise stated. All solutions were prepared using 18 MΩ·cm ultrapure Milli-Q water (MQ; Merck-Millipore, Bayswater, Australia). All glassware was acid washed in 5% HCl (v/v) for at least 1 week, then thoroughly rinsed in MQ before use. Stock solutions were stored in amber glass bottles at 4°C prior to use. To mimic the intracellular environment and focus on the effect of pH on iron catalyzed oxidation of DA, all experiments were performed in a light-free environment at a controlled temperature of 22 ± 0.6°C. This temperature, rather than a physiological temperature of 37°C was selected to allow direct comparison with previous work at pH 7.4. Investigation of the effect of temperature on the processes of interest in this work would be worthwhile though it should be recognized that thermodynamic and kinetic data for many of the key reactions underpinning these processes has been obtained by other investigators in the temperature range 20–25°C. Details of the preparation of stock and working solutions can be found in Supplementary Materials.

#### Preparation of buffer solutions

**Figure 1:**
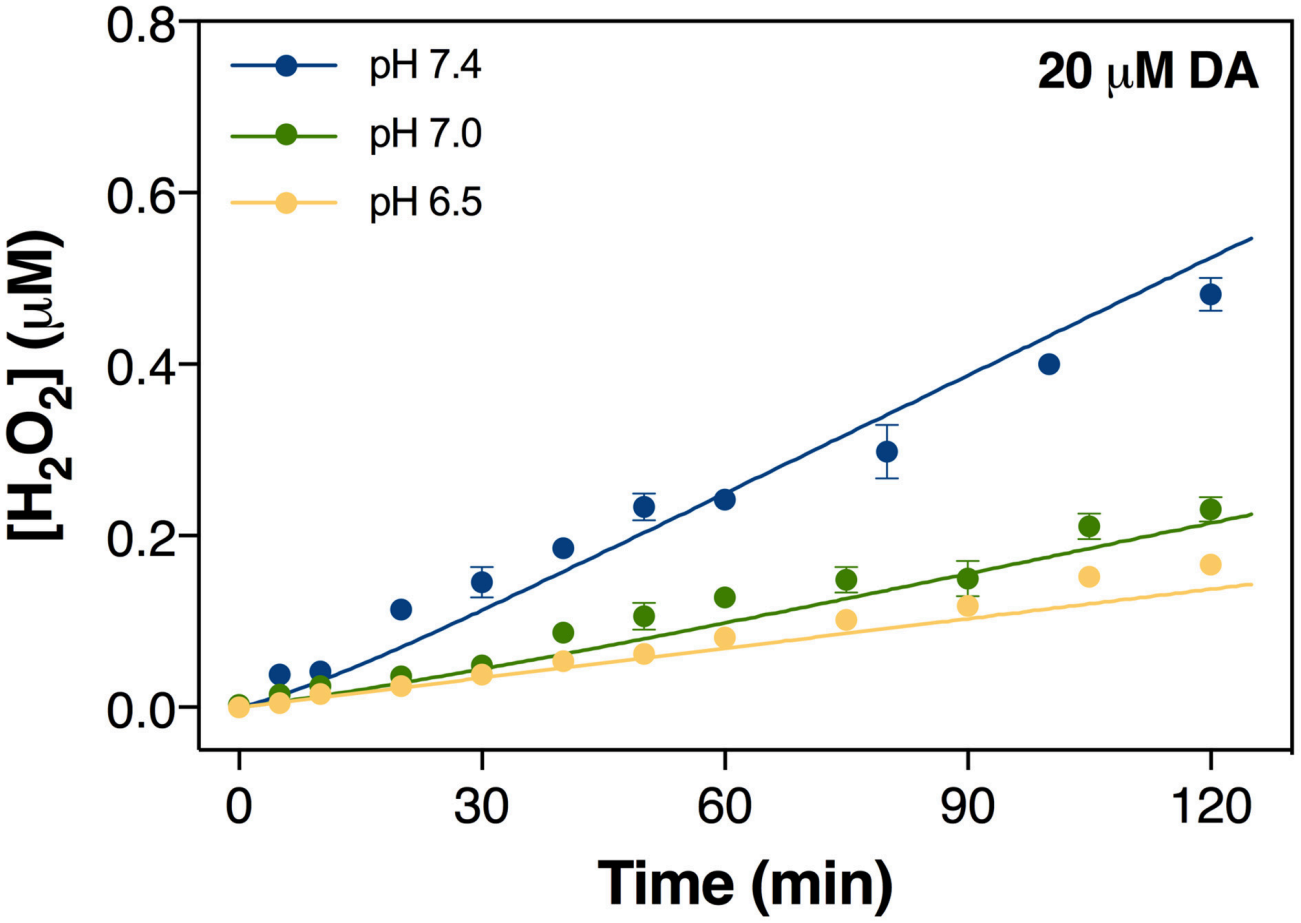
Formation of H_2_O_2_ *via* the oxidation of 20 μM DA in the absence of added iron at pH 6.5, pH 7.0, and pH 7.4 in 0.1 M NaCl solution. Error bars are standard errors from triplicate measurements and solid lines represent the model fit.

**Figure 2:**
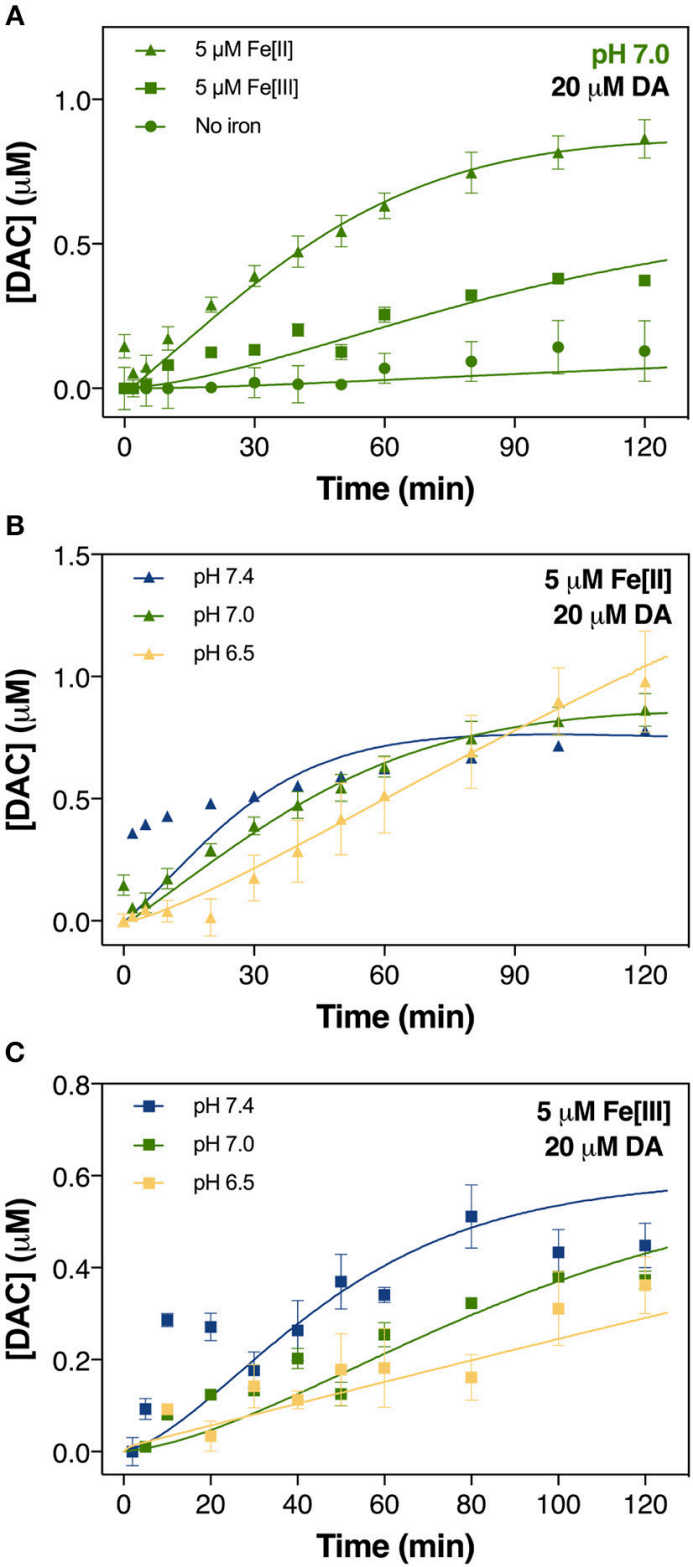
Formation of DAC in air-saturated (21% O_2_) 0.1 M NaCl solutions containing 20 μM DA and 5 μM iron (both Fe[III] and Fe[II]) at pH 7.0 **(A)**, in solutions containing 20 μM DA and 5 μM Fe[II] **(B)** and 5 μM Fe[III] **(C)** at pH 6.5, pH 7.0, and pH 7.4. Error bars are standard errors from triplicate measurements and solid lines represent the model fit.

**Figure 3:**
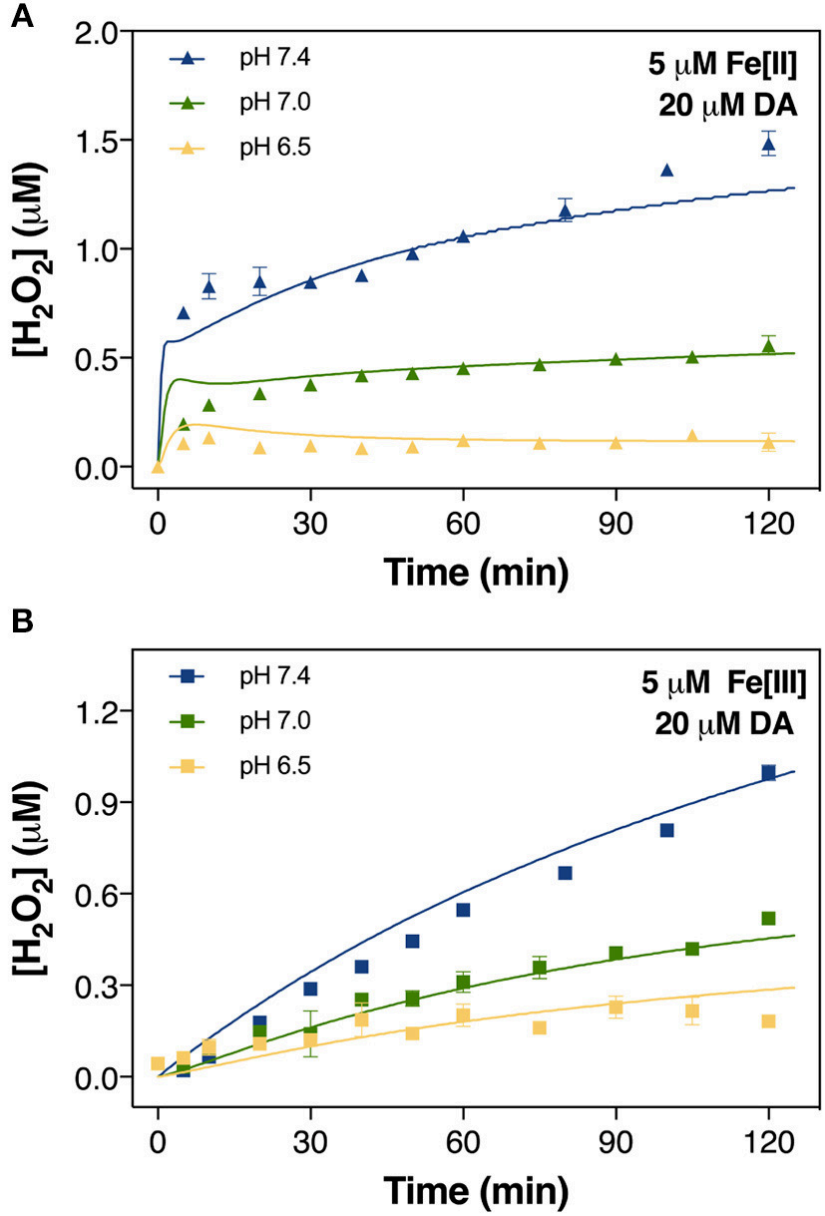
Formation of H_2_O_2_ in air-saturated (21% O_2_) 0.1 M NaCl solutions containing 20 μM DA and 5 μM Fe[II] **(A)** and 5 μM Fe[III] **(B)** at pH 6.5, pH 7.0, and pH 7.4. Error bars are standard errors from triplicate measurements and solid lines represent the model fit.

**Figure 4:**
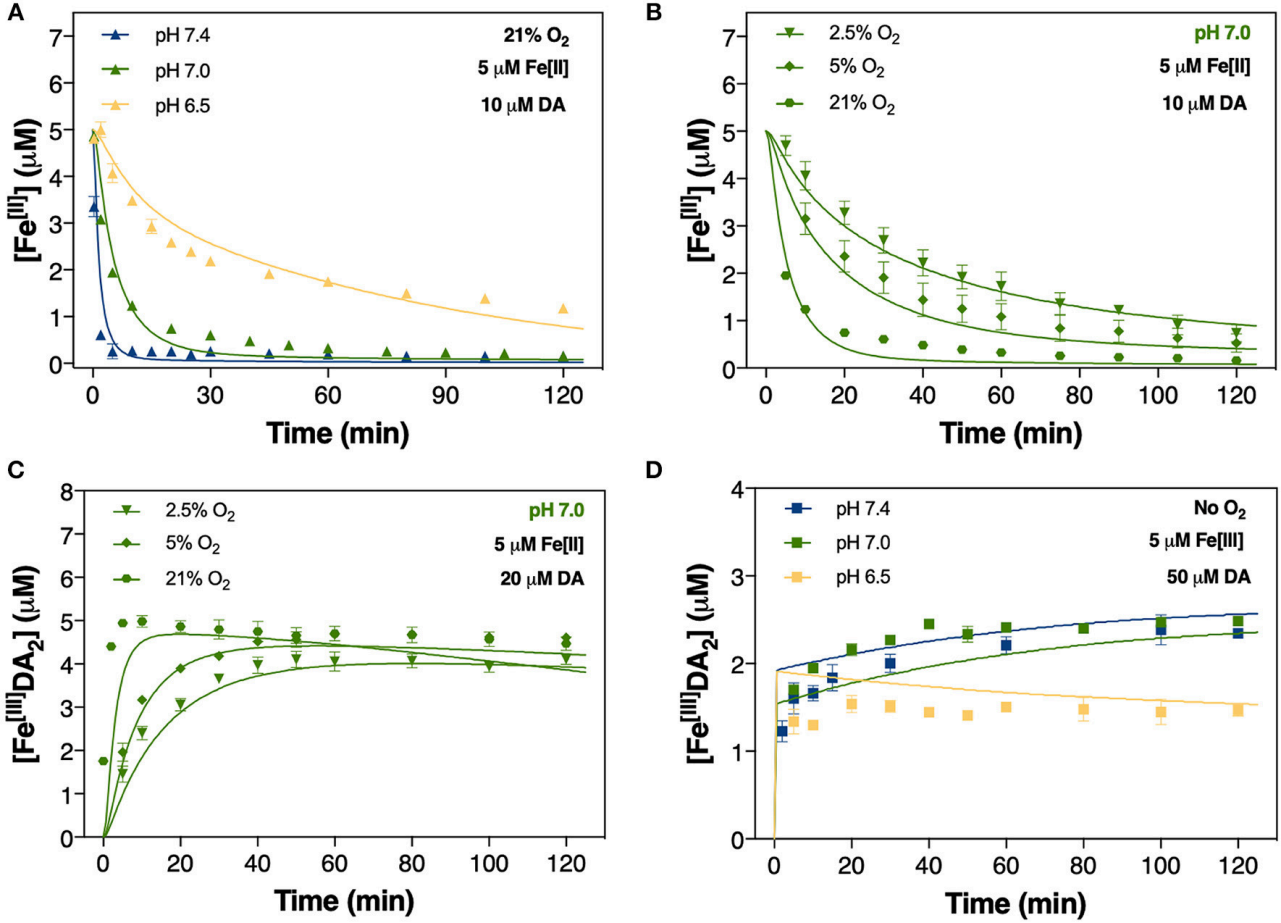
Oxidation of 5 μM Fe[II] at pH 6.5, pH 7.0, and pH 7.4 in air-saturated (21% O_2_) 0.1 M NaCl solutions containing 10 μM DA **(A)**; oxidation of 5 μM Fe[II] at pH 7.0 in the presence of 2.5, 5, and 21% O_2_ **(B)**; and the formation of Fe^III^DA_2_ **(C)** in 0.1 M NaCl solutions in the same O_2_ conditions. Formation of Fe^III^DA_2_ in 0.1 M deoxygenated NaCl solutions containing 5 μM Fe[III] and 50 μM DA at pH 6.5, pH 7.0, and pH 7.4 **(D)**. Error bars are standard errors from triplicate measurements and solid lines represent the model fit. The experimental data at pH 7.4 was taken from Sun et al. (2016).

In general, despite often being considered “chemically inert” (Yu et al., 1997; Thiel et al., 1998), buffers such as 3-(N-morpholino)propanesulfonic acid (MOPS), 2-(N-morpholino)ethanesulfonic acid (MES) and 4-(2-hydroxyethyl)piperazine-1-ethanesulfonic acid (HEPES) may still exert an influence on the spectroscopic measurement and/or formation of different target substances. As such, to eliminate any buffer induced experimental artifacts, studies were undertaken in 0.1 M NaCl, 2 mM NaHCO_3_ solutions containing 10 mM of MOPS, MES or HEPES by taking into consideration their pH control range and the influence they may exert on the measurement. Specifically, MOPS was used for the measurement of the generation of Fe^[III]^DA_2_, DAC and H_2_O_2_ over pH 6.5–7.4 (Figures 1–4 and Supplementary Figure 5) as it does not exhibit spectrum broadening effects. For the measurement of Fe[II], MES was used at pH 6.5, while HEPES was used at both pH 7.0 and 7.4 (Figure 4 and Supplementary Figure 7) as a result of the pH control range of these buffers and the negligible buffer facilitated reduction of Fe[III] in the presence of ferrozine (FZ; 4-[3-pyridin-2-yl-5-(4-sulfophenyl)-1,2,4-triazin-6-yl]benzenesulfonate) at pHs below 7.0.

#### Control of dissolved O_2_ content in buffer solutions

For experiments using variable O_2_ concentrations (0, 2.5, and 5% dissolved O_2_), buffer solutions prepared above were sparged with special gas mixtures (297 ± 6 ppm CO_2_ with or without 5% O_2_, in Ar; BOC Gases, Preston, Australia) for at least 1 h before the addition of DA and Fe (see Control of Dissolved O_2_ Content in Buffer Solutions). Constant O_2_ concentration was maintained by continuous sparging with the gas mixture over the course of the entire experiment.

### Analytical methods

#### pH measurements

All pH measurements were made using a Hanna Instruments HI9025 pH meter (Keysborough, Australia) with a glass electrode and Ag-AgCl reference electrode. The pH meter and electrode were calibrated prior to each experiment using NIST-traceable buffer solutions (pH 4.01, 7.01, and 10.01).

#### Ferrous iron quantification

Quantitative measurements of Fe[II] were made using the modified FZ method as the reduction of DA bound iron occurs in the presence of FZ at low pH (Garg et al., 2013). The UV absorbance of Fe^[II]^FZ_3_ (both from inorganically and organically bound Fe[II]) was monitored at 562 nm with baseline correction at 690 nm. The concentration of total Fe[II] was then calculated per the following (Garg et al., 2013):

$$\begin{array}{r} \left[\text{Fe}\left[\text{II}\right]\right]\;=\;\frac{\left(A_{562}-\;\epsilon_{\text{Fe}\left[\text{III}\right]}\;\times \;{\left[\text{Fe}\right]}_{T}\;\times \;l\right)}{\left(\varepsilon_{\text{Fe}\left[\text{II}\right]}-\;\varepsilon_{\text{Fe}\left[\text{III}\right]}\right)\;\times \;l} \end{array}$$

where *A*_562_ is the recorded absorbance at 562 nm, ε_Fe[II]_ and ε_Fe[III]_ are the molar absorption coefficients of the Fe^[II]^FZ complex at 562 nm arising from the presence of Fe[II] and Fe[III], respectively. [Fe]_*T*_ is the total iron concentration, and *l* is the path length (10 cm).

#### Iron-dopamine complex measurements

The concentration of Fe^[III]^DA_2_ was determined spectrophotometrically by measuring the absorbance at 580 nm with baseline correction at 850 nm. Calibration curves for quantification of the concentration of Fe^[III]^DA_2_ were constructed from measurements performed under anoxic conditions (Sun et al., 2016). Given the low concentrations and weak absorptivity of the *mono*-complex, the effect of this species on the measurement was considered negligible. It should be noted, however, that the Fe^[III]^DA complex is a precursor to the formation of the highly neurotoxic 6-OHDA quinone (Hare and Double, 2016). The molar absorptivity of Fe^[III]^DA_2_ was determined to be 3,121 M^−1^ cm^−1^ which was within 7% of the previously published value (Sever and Wilker, 2004). As a result of the slightly acidic pH and DA concentration used in this study, the extent of formation of the *tris*-complex should be negligible (Kowalchyk et al., 1995; Sun et al., 2018a). Even though the spectrum of DAC can overlap with that of the Fe^[III]^DA_2_ complex at 580 nm, the influence of DAC was not considered given the low concentration present (nominally <1 μM) under all conditions investigated herein (Pezzella et al., 1997) and the small molar absorptivity of DAC at 580 nm (ε_580_ = 439 M^−1^cm^−1^).

#### Hydrogen peroxide measurements

The H_2_O_2_ formed from Fe-mediated DA oxidation was quantified using the modified DPD method (Bader et al., 1988; Voelker and Sulzberger, 1996; Sun et al., 2016). Briefly, 1 mM DTPA was added to quench H_2_O_2_ generation at each time point in each assay (Sun et al., 2016). To eliminate interference from subsequent H_2_O_2_ generation and/or consumption as a result of the presence of high Fe[II] concentrations, 500 μM BPY was added prior to DTPA addition (Voelker and Sulzberger, 1996).

Quantitative data were interpolated by linear regression analysis of absorbance at 551 nm by increasing concentrations of H_2_O_2_ in 0.1 M NaCl with 60 μM DPD and 500 U/L HRP added, including a standard blank. No apparent interferences arising from the presence of moderate Fe[III] (5 μM) and DA (30 μM) were identified (Supplementary Figure 1). In order to enhance the accuracy of the measurements and remove potential interference arising from DA residuals on H_2_O_2_ absorbance measurements, the corresponding amount of DA, which was exactly the same as that used in a specific experiment set, was added to develop calibration curves for the experiments containing high concentrations of Fe[II].

#### Aminochrome quantification

The concentration of DAC was determined by measuring absorbance at 475 nm with baseline correction at 850 nm (Supplementary Figure 2; Herlinger et al., 1995; Pham and Waite, 2014). The molar absorptivity derived from the calibration curves indicated that the precursor DAQ was unlikely to exert any influence on the measurement of DAC while most phenolic organics, including DA (Supplementary Figure 3) and 5,6-dyhydroxyindole (DHI), only absorb at around 280 nm (Il'ichev and Simon, 2003). As such, the influence arising from the rearrangement products of DAC should be minimal at 475 nm. Absorbance at a particular wavelength is the sum of the absorbances contributed from different species, thus spectral overlap may cause false-positive measurements. Given the coexistence of Fe^[III]^DA_2_ and DAC in solutions containing iron and DA, the concentration of each species was determined by solving the linear equations as described previously (Sun et al., 2018b); i.e.,

$$A_{475}=\;\left(\varepsilon_{475}^{{\text{Fe}}^{\left[\text{III}\right]}{\text{DA}}_{2}}{\text{C}}_{{\text{Fe}}^{\left[\text{III}\right]}{\text{DA}}_{2}}\text{l}\right)+\;\left(\varepsilon_{475}^{\text{DAC}}C_{\text{DAC}}l\right)$$

where *A* is the total absorbance at 475 nm, $\varepsilon_{i}^{j}$ is the molar absorptivity of species *j* at wavelength *i, C*_*j*_ is the concentration of species *j* and *l* is the pathlength (10 cm). The concentration of Fe^[III]^DA_2_ was initially quantified based on the absorbance at 580 nm as DAC absorbed negligibly at this wavelength.

Calibration curves of DAC were developed by adding different concentrations of the freshly prepared DAC working solution into the air-saturated MOPS buffer solutions at pH 7.0 (Sun et al., 2018a). The molar absorptivity of DAC calculated in this study (ε_475__nm_ = 3,245 M^−1^cm^−1^) is similar to that reported by Segura-Aguilar and Lind (1989) (ε_475_ = 3,085 M^−1^cm^−1^) and Pham and Waite (2014) (ε_475_ = 3,281 M^−1^cm^−1^).

### Speciation and kinetic modeling

The pH-dependent distributions of DA, Fe[III] and Fe[II] species were determined using the program Visual Minteq (Gustafsson, 2005). Details of the distributions are shown in Supplementary Figure 4 with stability constants used in this study provided in Supplementary Table 1.

The kinetic model developed to describe the experimental data at pH 6.5, 7.0, and 7.4 was implemented using the software Kintek Explorer (Johnson et al., 2009). Specifically, the kinetic model is a set of reactions describing the key processes involved in the interplay between Fe and DA. To apply the model for the quantification of the time-dependent transformation of reactants, intermediates and products, the rate constant for each key process was either adopted from previous work or fitted in this study. The “goodness of fit” was judged by the ability of the reaction set used (and the associated set of coupled differential equations representing the rate expressions for each reaction) to describe the time-dependent transformation of a range of substances. If the model provided a poor description of the data, it indicated either a flaw in the reaction set or rate constant(s) used. In addition to the data collected in the current work, portions of the experimental data collected at pH 7.4 described by Sun et al. (2016) were used to complement this dataset and fully elucidate the effect of pH on DA oxidation. Given the critical role of dissociation of DA bound Fe at lower pH and the improved constraints provided by analysis of DAC, slight amendments were applied to the kinetic model previously developed for pH 7.4 (Sun et al., 2016).

## Results

### Development and rationale of experimental model

Given the relatively complicated model developed in this study, several intermediates were measured in order to better constrain the rate constants used herein. Briefly, (i) generation of DAC and H_2_O_2_ (shown in Figures 1–3) are used to constrain the rate constants for transformation of DA both in the absence and presence of iron including those for the oxidation of DA and leukoaminochrome (DAL) and cyclization of DAQ; (ii) decay of Fe[II] and formation of Fe-DA complexes in the presence of O_2_ (shown in Figures 4A–C) are used to constrain the rate constant for the DA-induced transformation of Fe; and (iii) formation of Fe[III]-DA complexes (shown in Figure 4D and Supplementary Figure 5) in the absence of O_2_ is used to constrain the rate constants for *mono*-complex formation, DA-induced reductive dissolution of ferrihydrite (amorphous ferric oxyhydroxide; or AFO) as well as the dynamic equilibrium between the *mono*- and *bis*-complexes. Details of the various reactions hypothesized to play a role are presented in Tables 1–3. Sensitivity analysis was used to determine the relative importance of the proposed reactions. Specifically, the greater the variation of the relative residuals that occurred with the change in magnitude of the rate constant, the more influential the reaction is. The lowest point or the “shift point” shown in the sensitivity analysis represents the optimal rate constant for the overall model applied. To simplify the applied model, rate constants were maintained consistent with those deduced in our previous study (Sun et al., 2016) with the exception of those with significant sensitivity to change of pH.

**Table 1:** Modeled reactions and rate constants for the autoxidation of DA at pH 6.5, 7.0, and 7.4.

	**No. reactions**	**Rate constants (M**^**−1**^**s**^**−1**^ **or s**^**−1**^**)**			**References**
		**pH 6.5**	**pH 7.0**	**pH 7.4**
1	$\text{DA}+\;{\text{O}}_{\text{2}}\;\overset{k_{1}}{\underset{}{\to }}{\text{O}}_{2}^{•-}\;+\text{D}{\text{A}}^{•-}$	*k*_1_ = 3.94 × 10^−3^	*k*_1_ = 4.22 × 10^−3^	*k*_1_ = 8.24 × 10^−3a^	This study
2	$\text{D}{\text{A}}^{•-}\;+\;{\text{O}}_{\text{2}}\underset{k_{-2}}{\overset{k_{2}}{⇌}}\;{\text{O}}_{2}^{•-}\;+\text{DAQ}$	*k*_2_ = 2.95 × 10^3^			1
		*k*_−2_ = 1.0 × 10^9^			1
3	$\text{D}{\text{A}}^{•-}\;+\text{D}{\text{A}}^{•-}\;\overset{k_{3}}{\underset{}{\to }}\text{DA}+\text{DAQ}$	*k*_3_ = 2.35 × 10^8^			2
4	$\text{DAQ}\overset{k_{4}}{\underset{}{\to }}\text{DAL}$	*k*_4_ = 1.23 × 10^−2^	*k*_4_ = 1.0	*k*_4_ = 4.45^a^	This study
5	$\text{DAL}+\text{DAQ}\overset{k_{5}}{\underset{}{\to }}\text{DA}+\text{DAC}$		*k*_5_ = 5.30 × 10^6^		3
6	$\text{DAL}+\;{\text{O}}_{\text{2}}\;\overset{k_{6}}{\underset{}{\to }}\text{DAC}+\;{\text{H}}_{\text{2}}{\text{O}}_{\text{2}}$	*k*_6_ = 1.17	*k*_6_ = 1.31	*k*_6_ = 5.12^a^	This study
7	${\text{O}}_{2}^{•-}\;+\;{\text{O}}_{2}^{•-}\;\overset{k_{7}}{\underset{}{\to }}\;{\text{H}}_{\text{2}}{\text{O}}_{\text{2}}\;+\;{\text{O}}_{\text{2}}$	*k*_7_ = 1.90 × 10^6^	*k*_7_ = 6.0 × 10^5^	*k*_7_ = 1.90 × 10^5^	4
8	$\text{D}{\text{A}}^{•-}\;+\;{\text{O}}_{2}^{•-}\;\overset{k_{8}}{\underset{}{\to }}\text{DAQ}+\;{\text{H}}_{\text{2}}{\text{O}}_{\text{2}}$		*k*_8_ = 8.27 × 10^9^^b^		5
9	$\text{DAC}\overset{k_{9}}{\underset{\text{Iron}}{\to }}{\text{Decay products}}^{\text{c}}$	*k*_9_ = 1.2 × 10^−6^	*k*_9_ = 8 × 10^−5^	*k*_9_ = 4 × 10^−4^	This study

a *Value modified from that used in Sun et al. (2016)*.

b *Rate constant taken from Sun et al. (2016)*.

c The rate constant proposed for reaction 9 incorporates the influence of Fe. DA, dopamine;

*DA^•−^, semiquinone radical; DAQ, dopamine-o-quinone; DAC, aminochrome; DAL, leukoaminochrome; and O${}_{2}^{•-}$, superoxide. Refs: (1) Pham and Waite (2014); (2) Borovansky et al. (2006); (3) Land et al. (2003); (4) Zafiriou (1990) and (5) Sun et al. (2016)*.

**Table 2:** Modeled reactions and rate constants for Fe[III]-catalyzed oxidation of DA at pH 6.5,7.0 and 7.4.

	**No. reactions**	**Rate constants (M**^**−1**^**s**^**−1**^ **or s**^**−1**^**)**			**References**
		**pH 6.5**	**pH 7.0**	**pH 7.4**
10	$\text{Fe}\left[\text{III}\right]\;+\text{Fe}\left[\text{III}\right]\;\overset{k_{10}}{\underset{}{\to }}\text{AFO}+\text{n}{\text{H}}^{\text{+}}$	*k*_10_ = 1.0 × 10^6^	*k*_10_ = 3.4 × 10^6^	*k*_10_ = 5.0 × 10^6^	6
11	$>\text{Fe[III}{\text{]}}_{\text{n}}\;+\text{DA}\overset{k_{11}}{\underset{}{\to }}\text{> Fe[III}{\text{]}}_{\text{n – 1}}\;+\text{F}{\text{e}}^{\text{[III]}}\text{DA}$	*k*_11_ = 0.599	*k*_11_ = 2.3	*k*_11_ = 2.34^b^	This study
12	$>\text{Fe[III}{\text{]}}_{\text{n}}\;+\text{DA}\overset{k_{12}}{\underset{}{\to }}\text{> Fe[III}{\text{]}}_{\text{n – 1}}\;+\text{Fe[II] + D}{\text{A}}^{•-}$	*k*_12_ = 0.008	*k*_12_ = 0.3	*k*_12_ = 0.6^b^	This study
13	$\text{Fe[III]}+\text{DA}\underset{k_{-13}}{\overset{k_{13}}{⇌}}\text{F}{\text{e}}^{\text{[III]}}\text{DA}$	*k*_13_ = 8.7 × 10^4^	*k*_13_ = 2.09 × 10^5^	*k*_13_ = 4.15 × 10^5^^a^	This study
		*k*_−13_ = 1.45	*k*_−13_ = 0.969	*k*_−13_ = 0.463	This study
14	$\text{F}{\text{e}}^{\text{[III]}}\text{DA}+\text{DA}\underset{k_{-14}}{\overset{k_{14}}{⇌}}\text{F}{\text{e}}^{\text{[III]}}\text{D}{\text{A}}_{\text{2}}$	*k*_14_ = 4.50 × 10^5^			7
		*k*_−14_ = 3.37 × 10^−4^	*k*_−14_ = 2.86 × 10^−4^	*k*_−14_ = 2.59 × 10^−4^	This study
15	$\text{F}{\text{e}}^{\;\left[\text{III}\right]\;}\text{DA}+{\text{O}}_{2}^{•-}\;\overset{k_{15}}{\underset{}{\to }}\text{F}{\text{e}}^{\;\left[\text{II}\right]\;}\text{DA}+\;{\text{O}}_{\text{2}}$	*k*_15_ = 1.50 × 10^8^			8
16	$\text{F}{\text{e}}^{\;\left[\text{III}\right]\;}\text{DA}\overset{k_{16}}{\underset{}{\to }}\text{Fe}\left[\text{II}\right]\;+\text{D}{\text{A}}^{•-}$	*k*_16_ = 0.23			9
17	$\text{F}{\text{e}}^{\;\left[\text{III}\right]\;}\text{D}{\text{A}}_{\text{2}}\;\overset{k_{17}}{\underset{}{\to }}\text{Fe}\left[\text{II}\right]\;+\text{DA}+\text{D}{\text{A}}^{•-}$	*k*_17_ = 7.26 × 10^−5^^b^			5
18	$\text{Fe}\left[\text{III}\right]{+\text{O}}_{2}^{•-}\;\overset{k_{18}}{\underset{}{\to }}\text{Fe}\left[\text{II}\right]\text{+}{\text{O}}_{\text{2}}$	*k*_18_ = 1.50 × 10^8^			10
19	$>\text{Fe[III}{\text{]}}_{\text{n}}\;+{\text{O}}_{2}^{•-}\;\overset{k_{19}}{\underset{}{\to }}>\text{Fe[III}{\text{]}}_{\text{n}-1}\;+\text{Fe[II] +}{\text{O}}_{2}$	*k*_19_ = 4.84 × 10^4^	*k*_19_ = 3.70 × 10^5^	*k*_19_ = 3.70 × 10^5^^b^	This study
20	$\text{Fe}\left[\text{III}\right]\;+\text{D}{\text{A}}^{•-}\;\overset{k_{20}}{\underset{}{\to }}\text{Fe}\left[\text{II}\right]\;+\text{DAQ}$	*k*_20_ = 9.12 × 10^9^			This study

a *Modified value of the model developed at pH 7.4 in Sun et al. (2016)*.

b *Rate constant taken from Sun et al. (2016)*.

*DA, dopamine; DA^•−^, dopamine semiquinone radical; DAQ, dopamine-o-quinone; $O_{\text{2}}^{•-}$, superoxide; Fe[III], inorganic ferric ion; Fe[III]_I_, total inorganic Fe[III]; AFO, ferrihydrite; and Fe[II], inorganic ferrous ion. Refs: (6) Pham et al. (2006); (7) Blesa and Matijevi (1989); (8) Rose and Waite (2003); (9) El-Avaan et al. (1997) and (10) Rush and Bielski (1985)*.

**Table 3:** Modeled reactions and rate constants for Fe[II]-catalyzed oxidation of DA at pH 6.5, 7.0 and 7.4.

	**No. reactions**	**Rate constants (M**^**−1**^**s**^**−1**^ **or s**^**−1**^**)**			**References**
		**pH 6.5**	**pH 7.0**	**pH 7.4**
21	$\text{Fe}\left[\text{II}\right]\;+\;{\text{O}}_{\text{2}}\;\overset{k_{21}}{\underset{}{\to }}\text{Fe}\left[\text{III}\right]\;+{\text{O}}_{2}^{•-}$	*k*_21_ = 0.0209	*k*_21_ = 0.0959	*k*_21_ = 0.77^b^	This study
22	$\text{Fe}\left[\text{II}\right]\;+{\text{O}}_{2}^{•-}\;\overset{k_{22}}{\underset{}{\to }}\text{Fe}\left[\text{III}\right]\;+\;{\text{H}}_{\text{2}}{\text{O}}_{\text{2}}$	*k*_22_ = 1.0 × 10^7^			10
23	$\text{Fe}\left[\text{II}\right]\;+\;{\text{H}}_{\text{2}}{\text{O}}_{\text{2}}\;\overset{k_{23}}{\underset{}{\to }}\text{Fe}\left[\text{III}\right]\;+{\;}^{•}\text{OH}+\text{O}{\text{H}}^{\text{–}}$	*k*_23_ = 1.72 × 10^3^	*k*_23_ = 4.79 × 10^3^	*k*_23_ = 1.33 × 10^4^	11
24	$\text{Fe[II]}+\text{DA}\underset{k_{-24}}{\overset{k_{24}}{⇌}}\text{F}{\text{e}}^{\text{[II]}}\text{DA}$	*k*_24_ = 7.0 × 10^2^	*k*_24_ = 7.2 × 10^2^	*k*_24_ = 7.5 × 10^2^^b^	This study
		*k*_−24_ = 1.02 × 10^−2^	*k*_−24_ = 9.2 × 10^−3^	*k*_−24_ = 1.6 × 10^−3^	This study
25	$\text{F}{\text{e}}^{\;\left[\text{II}\right]\;}\text{DA}+\;{\text{O}}_{\text{2}}\;\overset{k_{25}}{\underset{}{\to }}\text{F}{\text{e}}^{\;\left[\text{III}\right]\;}\text{DA}+{\text{O}}_{2}^{•-}$	*k*_25_ = 3.32	*k*_25_ = 19	*k*_25_ = 145^b^	This study
26	$\text{F}{\text{e}}^{\;\left[\text{II}\right]\;}\text{DA}+\;{\text{H}}_{\text{2}}{\text{O}}_{\text{2}}\;\overset{k_{26}}{\underset{}{\to }}\text{F}{\text{e}}^{\;\left[\text{III}\right]\;}\text{DA}+{\;}^{•}\text{OH}+\text{O}{\text{H}}^{\text{–}}$	*k*_26_ = 1.72 × 10^3^	*k*_26_ = 4.79 × 10^3^	*k*_26_ = 1.33 × 10^4^	11
27	$\text{F}{\text{e}}^{\;\left[\text{II}\right]\;}\text{DA}+{\text{O}}_{2}^{•-}\;\overset{k_{27}}{\underset{}{\to }}\text{F}{\text{e}}^{\;\left[\text{III}\right]\;}\text{DA}+\;{\text{H}}_{\text{2}}{\text{O}}_{\text{2}}$	*k*_27_ = 1.0 × 10^7^			8
28	$\text{F}{\text{e}}^{\;\left[\text{II}\right]\;}\text{DA}+\text{D}{\text{A}}^{•-}\;\overset{k_{28}}{\underset{}{\to }}\text{F}{\text{e}}^{\;\left[\text{III}\right]\;}\text{DA}+\text{DA}$	*k*_28_ = 1.92 × 10^5^^b^			5
29	$\text{Fe}\left[\text{II}\right]\;+\text{D}{\text{A}}^{•-}\;\overset{k_{29}}{\underset{}{\to }}\text{F}{\text{e}}^{\;\left[\text{III}\right]\;}\text{DA}+\text{DA}$	*k*_29_ = 1.92 × 10^5^^b^			5

a *Modified value of the model developed at pH 7.4 in Sun et al. (2016)*.

b *rate constant taken from Sun et al. (2016)*.

*DA, dopamine; DA^•−^, dopamine semiquinone radical; O${}_{2}^{•-}$, superoxide; Fe[III], inorganic ferric ion; Fe[II], inorganic ferrous ion; H_2_O_2_, hydrogen peroxide; and ^•^OH, hydroxyl radical. Refs: (11) González-Davila et al. (2005)*.

The sensitivity of the model to changes in individual rate constant values, defined as the relative residual, *r*, was determined using the program Kintecus (Ianni, 2003) combined with a Visual Basic for Applications (VBA) program. Note that the relative residual is defined as:

$$\begin{array}{r} r\;=\;\frac{1}{n}\sum_{i=1}^{n}\frac{\left|MP_{i}-ED_{i}\right|}{ED_{i}} \end{array}$$

where *MP*_*i*_ is the model prediction, *ED*_*i*_ is the experimental data at the same condition and time interval and *n* represents the total number of measured data points.

As shown in Supplementary Figures 6A,B, significant influence of DAQ cyclization and DAC decay on the transformation of DA was observed at the two pH values investigated here (the results of previous studies at pH 7.4 are provided by Sun et al., 2016). The most sensitive point of the relative residual *r* increases in line with pH. This is in agreement with the proposed rate constants (Tables 1–3) in the main text as deprotonation is generally the prerequisite for DAQ cyclization and DAC decay. In contrast, a relatively insensitive relative residual *r* is observed below the deduced upper rate constant (*k*_5_ = 5.3 × 10^6^) for the redox exchange between DAQ and DAL (Land et al., 2003; Sun et al., 2016), especially at pH 7.0 (Supplementary Figure 6C). As such, the rate constant of the redox exchange reaction at both pH 6.5 and 7.0 was consistent with these previously reported findings.

As shown in Supplementary Figure 6D, the DA-induced dissolutionanalysis shown in of AFO is an important process as the relative residual *r* is very sensitive to the change in the magnitude of the rate constant. A slight increase in the shift point with increase in pH is observed with this result in accord with the proposed rate constants applied. However, compared with that of DA-induced dissolution of AFO, the reductive dissolution after the adsorption of DA onto the surface of AFO (Reaction 12, Table 2) is relatively insensitive at both pH values used (Supplementary Figure 6E), indicating that reductive dissolution is not as important as DA-induced dissolution of AFO at each pH value investigated herein. As shown in Supplementary Figures 6F,G, the formation of both Fe^[III]^DA (Reaction 13, Table 2) and Fe^[II]^DA (Reaction 24, Table 3) are key reactions in this study as can be seen from the relative sensitivities of the relative residuals for these reactions. The rate constant for the formation of the *bis*-complex from Fe^[III]^DA with another DA molecule (Reaction 14, Table 2) is assumed to be similar to the rate constant for water-loss from Fe(OH)(H_2_O)${}_{5}^{2+}$ of 4.50 × 10^5^ M^−1^s^−1^ (Blesa and Matijevi, 1989) in view of the fact that the replacement of a coordinated H_2_O by the additional DA molecule is generally faster than the formation of the *mono*-complex (Ludwig et al., 1995). The results of sensitivity analysis shown in Supplementary Figure 6H indicate that this assigned value should be reasonable for the sensitivity of relative residual *r*. Similar to the formation of the Fe-DA complexes, dissociation of these complexes is also of great significance given the considerable sensitivity of the rate constants over several orders of magnitude (Supplementary Figures 6I–K). In contrast to increase in the optimal value on decrease in pH (Supplementary Figure 6I), the increase in the dissociation rate constant shown in Supplementary Figure 6J is much less significant. This supports the supposition that dissociation is generally important at low pH with the *bis*-complex much more stable in view of the iron sequestration. As shown in Supplementary Figure 6L, relative insensitivity of the relative residual *r* is evident for the deduced rate constant (3.7 × 10^5^ M^−1^s^−1^) for the reaction between superoxide and AFO (Reaction 19, Table 2). As such, in order to simplify the model, the rate constant of this reaction at high pH is considered to be the same as that proposed previously (Sun et al., 2016). However, a value for this rate constant of one order of magnitude lower is deduced from model fitting in this study at pH 6.5 with this lower value possibly a result of the increased proportion of HO${}_{2}^{•}$ at this lower pH and the subsequent reduced electrostatic attraction between superoxide and the AFO surface.

Theoretically, change in pH would typically result in variation of the Fe[II] oxidation rate in the presence of oxidants such as DA^•−^. However, as shown in Supplementary Figure 6M, considerable insensitivity of the relative residual is observed around the value proposed at physiological pH. As such, a consistent rate constant was used. Reduction of Fe[III] by DA^•−^ is important at pH 6.5 given the significant decrease in relative residuals on variation of the rate constant (from 1 to 10^10^ M^−1^s^−1^; shown in Supplementary Figure 6N). Therefore, the reaction between Fe[III] and DA^•−^ is considered in the proposed reaction scheme. The reduction of DA bound Fe[III] by DA^•−^ is not as significant as the reduction of Fe[III] in view of its lower reduction potential. As such, reduction of organically complexed Fe[III] by DA^•−^ is not considered in the reaction scheme.

### Dopamine-derived free radical production is pH dependent

To assess how pH influences the generation of toxic DA metabolites, the autoxidation of DA over a range of pH was initially investigated with attention given to the accumulation of H_2_O_2_ and DAC. As shown in Figure 1, spontaneous oxidation of DA is highly pH-dependent with 20 μM DA producing H_2_O_2_ at rates of 0.08, 0.12, and 0.24 μM h^−1^ at pH 6.5, 7.0, and 7.4, respectively. On the other hand, <0.1 μM of DAC was produced at pH 7.0 in the absence of Fe (Figure 2A).

The oxidation of DA by O_2_:

$$\begin{array}{r} \text{DA}+{\text{O}}_{2}\to \text{D}{\text{A}}^{•-}+{\text{O}}_{2}^{•-} \end{array}$$

is often considered to be a critical step in the production of H_2_O_2_ and DAC. Firstly, DA^•−^ can disproportionate to form DAQ which subsequently cyclizes to DAL. DAC and H_2_O_2_ can then be produced either through the redox exchange between DAQ and DAL (Equation 7) or as a result of the direct oxidation of DAL (Equation 8), O${}_{2}^{•-}$ disproportionation (Equation 9) and/or the O${}_{2}^{•-}$-mediated transformation of radicals such as DA^•−^ (Equation 10; Hawley et al., 1967; Graham, 1978; Sun et al., 2016).

$$\begin{array}{rcl} \text{DAQ}+\text{DAL} & \to & \text{DA}+\text{DAC} \end{array}$$

$$\begin{array}{rcl} \text{DAL}+{\text{O}}_{2} & \to & \text{DAC}+{\text{H}}_{2}{\text{O}}_{2} \end{array}$$

$${\text{O}}_{2}^{•-}+{\text{O}}_{2}^{•-}\to {\text{H}}_{2}{\text{O}}_{2}+{\text{O}}_{2}$$

$${\text{O}}_{2}^{•-}+{\text{DA}}^{•-}\to \text{DAQ}+{\text{H}}_{\text{2}}{\text{O}}_{\text{2}}$$

Among the various reactions shown above, the direct oxidation of DA by O_2_ to DA^•−^ and O${}_{2}^{•-}$ (Equation 6) is expected to be rate limiting in view of the spin restriction inherent between DA and O_2_ and, accordingly, exhibits a small rate constant (Table 1). With this in mind, any factors that influence this rate-limiting step (such as the presence of Fe[II] and Fe[III]; see Section Iron Accelerates Dopamine Oxidation) will alter the rate of formation of products arising from the oxidation of DA.

In general, the apparent oxidation rate of DA is determined by the contribution of different DA species. The pH-dependent formation of H_2_O_2_ shown in Figure 1 is attributed, at least partially, to the increase in the proportion of deprotonated DA ions on increase in pH; i.e.,

$$\begin{array}{r} k_{app}=\alpha_{{\text{H}}_{2}\text{DA}}k_{{\text{H}}_{2}\text{DA}+{\text{O}}_{2}}+{\text{α}}_{\text{HDA}}k_{\text{HD}{\text{A}}^{-}+{\text{O}}_{2}}\\ +{\text{α}}_{\text{D}{\text{A}}^{2-}}k_{\text{D}{\text{A}}^{2-}+{\text{O}}_{2}} \end{array}$$

where *k*_*app*_ is the apparent oxidation rate constant, α_*i*_ is the fraction of total DA species present as species *i* and *k*_*i*_ is the intrinsic oxidation rate constant of species *i*. Previous work has suggested that the abstraction of a hydrogen atom from the *mono*-deprotonated form of DA is the rate limiting step in the oxidation of DA (Herlinger et al., 1995). As such, it is not unexpected that an increase in the proportion of deprotonated DA on increase in pH would give rise to the enhanced generation of both DA^•−^ and O${}_{2}^{•-}$ *via* Equation 6, eventually resulting in the subsequent increase in the concentration of H_2_O_2_. Compared with the significantly enhanced production of H_2_O_2_ in the presence of iron (shown in section Iron Accelerates Dopamine Oxidation), while continuously formed, the toxicity induced by the autoxidation of DA is unlikely to be significant given the presence of *in vivo* oxidant removal enzymes, such as superoxide dismutase 1 (SOD1) and the glutathione peroxidases (GPx), though there is evidence that both enzymes are dysfunctional in Parkinson's disease (Cardoso et al., 2017; Trist et al., 2017, 2018). Additionally, the presence of DA-derived quinones is expected to be negligible (as shown in Figure 2A).

### Iron accelerates dopamine oxidation

In order to quantify the rate of generation of ROS and reactive quinones arising from the interaction between Fe and DA over pHs ranging from those of the physiological neuronal cytosol to those that have experienced disease-related and *post-mortem* acidosis, the accumulation of DAC and the concentrations of the key Fenton reagents–Fe[II] and H_2_O_2_-were subsequently investigated.

In contrast to the negligible production of DAC in the absence of Fe, a substantially higher concentration was generated in the presence of 5 μM iron at pH 7.0. In general, our results show that Fe[II] was more effective in catalyzing DAC production than was Fe[III] (Figure 2A). The initial rate of formation of DAC in the presence of both Fe[II] and Fe[III] increased significantly at pH 7.0 and 7.4 (Figures 2B,C). By contrast, the concentration of DAC plateaued in the latter stages of the 120 min assessment period at pH 7.4, more so in the presence of Fe[II].

Accordingly, labile Fe also accelerated the production of H_2_O_2_, again with Fe[II] being generally more efficient than Fe[III], particularly during the initial ~10 min following addition of reagents (Figure 3A). While H_2_O_2_ was continuously produced at pH 7.0 and 7.4 in the presence of both Fe species, at pH 6.5 only Fe[III] showed an increase in H_2_O_2_ production though the concentration present at *t* = 120 min was only ~10% of that produced at pH 7.4 (Figure 3B). For Fe[II], H_2_O_2_ concentration remained stable after a subtle increase from *t* = 0–10 min.

Accompanying the rapid generation of both DAC and H_2_O_2_ was a significant decrease in the concentration of Fe[II]. The apparent oxidation rate of Fe[II] significantly increased in the presence of DA. In the absence of DA, the concentration of Fe[II] halved over 2 h at pH 7.0 (Supplementary Figure 7A) while Fe[II] levels were near-totally depleted within 30 min in the presence of 10 μM DA at the same pH (Figure 4A).

According to the rate law:

$$\begin{array}{r} -\frac{d\left[\text{Fe}\left[\text{II}\right]\right]}{dt}\;=\;k\left[\text{Fe}\left[\text{II}\right]\right]\left[{\text{O}}_{2}\right] \end{array}$$

the oxidation of Fe[II] depends on the concentration of dissolved O_2_ present. As the O_2_ concentration in brain tissue is typically in the range of 10–60 μM (Koppenol and Butler, 1985; Ndubuizu and Lamanna, 2007), which corresponds to < 5% O_2_ saturation in aqueous solutions, the effect of O_2_ (at 2.5 and 5% saturation) on the transformation of Fe[II] in the presence of DA was examined at pH 7.0. As expected, both the rate and extent of Fe[II] oxidation decreased with lower O_2_ concentration, with only a 10% decrease in Fe[II] concentration at pH 7.0 in 5% O_2_ at *t* = 2 h compared with a 40% decrease at 21% O_2_ saturation (Supplementary Figure 7B). Oxidation of Fe[II] followed an exponential decay on addition of 10 μM DA at pH 7.0 with initial Fe[II] exhausted after ~70 min in 21% O_2_ and 80–90% loss at 2 h for physiologically-relevant O_2_ concentrations (Figure 4B). Accordingly, the initial formation of Fe^III^DA_2_ was strongly dependent on O_2_ concentration with markedly lower rates observed at 2.5 and 5% O_2_ (Figure 4C).

### Dopamine induces iron mobilization

As predicted by thermodynamic and kinetic data, the majority of Fe[III] should be present in an insoluble phase, even in the presence of 20 μM DA. As such, the continuous generation of both DAC and H_2_O_2_ in the presence of Fe[III] shown in Figures 2C, 3B suggests that DA is capable of inducing the slow release of reactive soluble Fe species. Indeed, dissolution of precipitated Fe by DA may pose a potential risk in view of its contribution to the labile iron pool (Dixon and Stockwell, 2014) and subsequent DA oxidation. Thus, to understand the extent of DA-induced mobilization of Fe[III], the temporal change in concentration of Fe^[III]^DA_2_ was measured over the pH range of 6.5 to 7.4. To eliminate potential confounding effects of O_2_-mediated transformation of both Fe and reducing radicals, Fe^[III]^DA_2_ formation was investigated under anoxic conditions. As shown in Figure 4D, Fe^[III]^DA_2_ formation increased gradually at pH 7.0 and 7.4 in the presence of high initial DA concentration (50 μM) though not at pH 6.5. At this pH 6.5, Fe^[III]^DA_2_ formation was ~60% of that produced at circumneutral pH; a trend conserved at a lower DA concentration (20 μM; Supplementary Figure 5).

Compared with pH 6.5, the increase in the overall concentration of Fe^III^DA_2_ at pH 7.0 and 7.4 can most likely be attributed to: (i) the reduced rate of LMCT resulting from the increased proportion of Fe^III^DA_2_ present, and (ii) more efficient formation of transient >Fe[III]-DA surface complexes that precede thermal detachment of DA-bound Fe (Equation 13) and/or DA-induced reductive dissolution (Equation 14):

$$\begin{array}{rcl} >{\text{Fe}\left[\text{III}\right]}_{\text{n}}+\text{DA} & \to & >{\text{Fe}\left[\text{III}\right]}_{\text{n}-1}+{\text{Fe}}^{\left[\text{III}\right]}\text{DA} \end{array}$$

$$\begin{array}{rcl} >{\text{Fe}\left[\text{III}\right]}_{\text{n}}+\text{DA} & \to & >{\text{Fe}\left[\text{III}\right]}_{\text{n}-1}+\text{Fe}\left[\text{II}\right]+{\text{DA}}^{•-} \end{array}$$

### *Ex vivo* modeling predicts potential toxicity of iron-mediated dopamine oxidation

**Figure 5:**
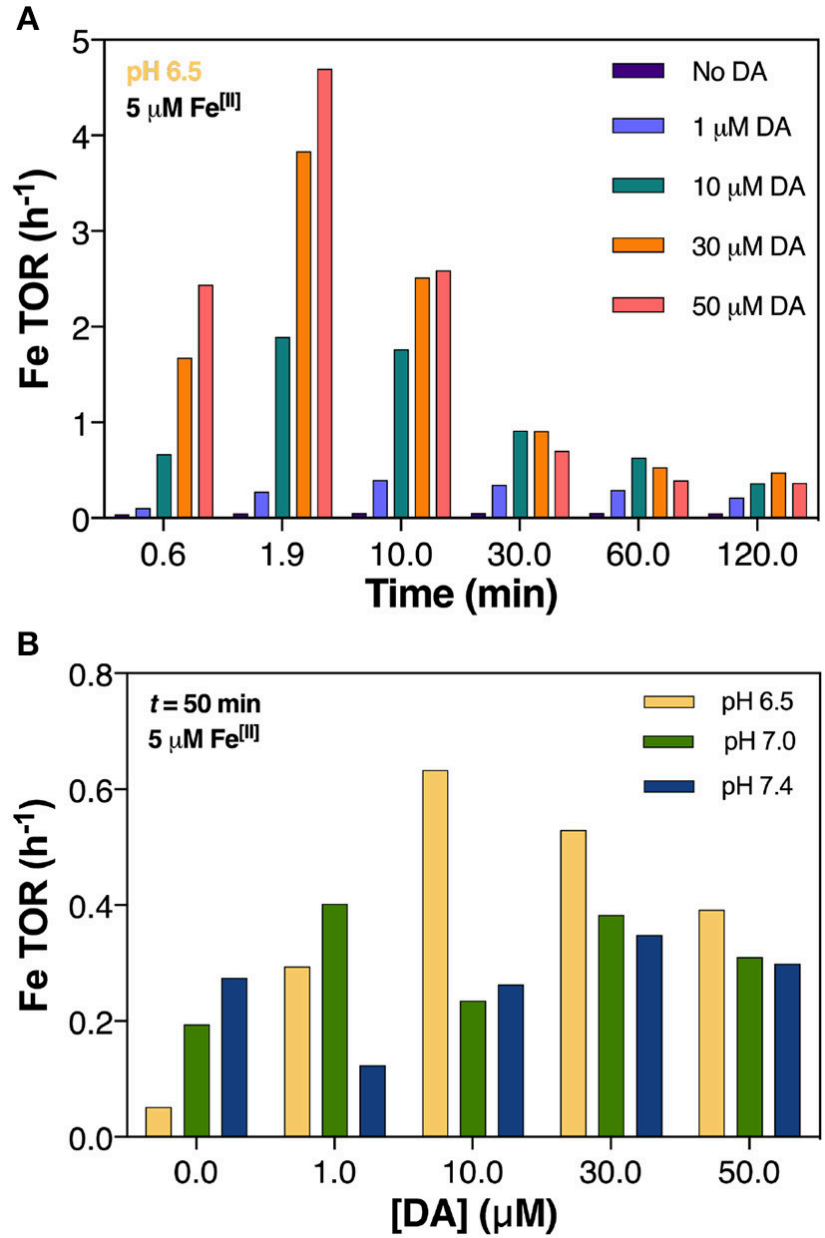
Predicted turnover rate (TOR) of 5 μM Fe[II] in air-saturated (21% O_2_) solutions with increasing DA concentrations at pH 7.0 **(A)** and at pH 6.5, pH 7.0, and pH 7.4 after 50 min **(B)**.

The kinetic model developed in this study was used to infer potential long-term effects of the combined mechanisms of DA oxidation in the presence of Fe. Particular emphasis was given to the effect of system conditions on Fe turnover rate (TOR; used as a measure of Fe-specific catalysis) and associated hydroxyl radical and DAC production, encompassing changing DA and H_2_O_2_ concentrations and steady-state dissolved O_2_. In this study, given the overall goodness of the model fit to the experimental data (Figures 1–5, Supplementary Figures 5, 7), the TOR was determined from the oxidation rate of Fe[II] as predicted by the model under particular conditions (e.g., pH, total concentration of Fe, the concentration ratio of Fe to DA and O_2_ content).

#### Dopamine concentration mediates iron redox cycling

In general, cycling rate of Fe was highest during the initial phase of each experiment and was amplified by increasing concentration of DA (Figure 5A) and higher pH (Supplementary Figure 9). After the initial dynamic cycling period, the TOR steadily decreased and appeared to reach equilibrium after ~30 min. Both acidic pH (pH 6.5) and DA concentration dictated higher Fe TOR at the near-midpoint of the reaction time (*t* = 50 min); the presence of 30 μM DA induced a 10-fold increase in turnover rate compared to DA-absent conditions at pH 6.5 whereas at pH 7.0 and 7.4 the increase in TOR represented only 2 and 1.3-fold change, respectively (Figure 5B). From this, we conclude that the effect of DA on Fe TOR is much more substantial in acidic conditions with this likely contributing to the abeyance of H_2_O_2_ generation (shown in Figure 3A) and predicted increase in ^•^OH production as a result of both Fenton chemistry (Equations 1, 2) and Fe[II]-mediated DA oxidation (Reactions 23 and 26, Table 3).

#### Kinetic modeling of sustained parkinsonian oxidative stress

As a result of the attenuation by endogenous antioxidants, including SOD1, GPx, and endogenous ascorbate ions (Harrison and May, 2009), *in vivo* steady-state concentrations of H_2_O_2_ stemming from normal mitochondrial respiration in the brain are estimated to be maintained at around 5 nM (Adam-Vizi, 2005). However, concomitant hypoxia thought to occur in the Parkinson's disease brain (Adams and Odunze, 1991) may result in both the accumulation of lactate as a result of the anaerobic conditions, which, in turn, may contribute to the decrease in pH, as well as a release of DA from chemically-isolated vesicles (Phebus et al., 1986).

To predict the rate of ^•^OH generation in a chemical environment more reflective of continued replenishment of pro-oxidants in a DA neuron, the kinetic model was applied where pseudo-equilibrium was reached in the presence of a range of fixed H_2_O_2_ and DA concentrations. While it must be emphasized that this model is illustrative, as it is specific to only DA-derived oxidative stress, it represents an important advance in understanding the biochemical mechanism by which gradual neuronal Fe accumulation with age can become pathological in cells with both high metabolic output and an abundance of a pro-oxidant catecholamine in DA.

**Figure 6:**
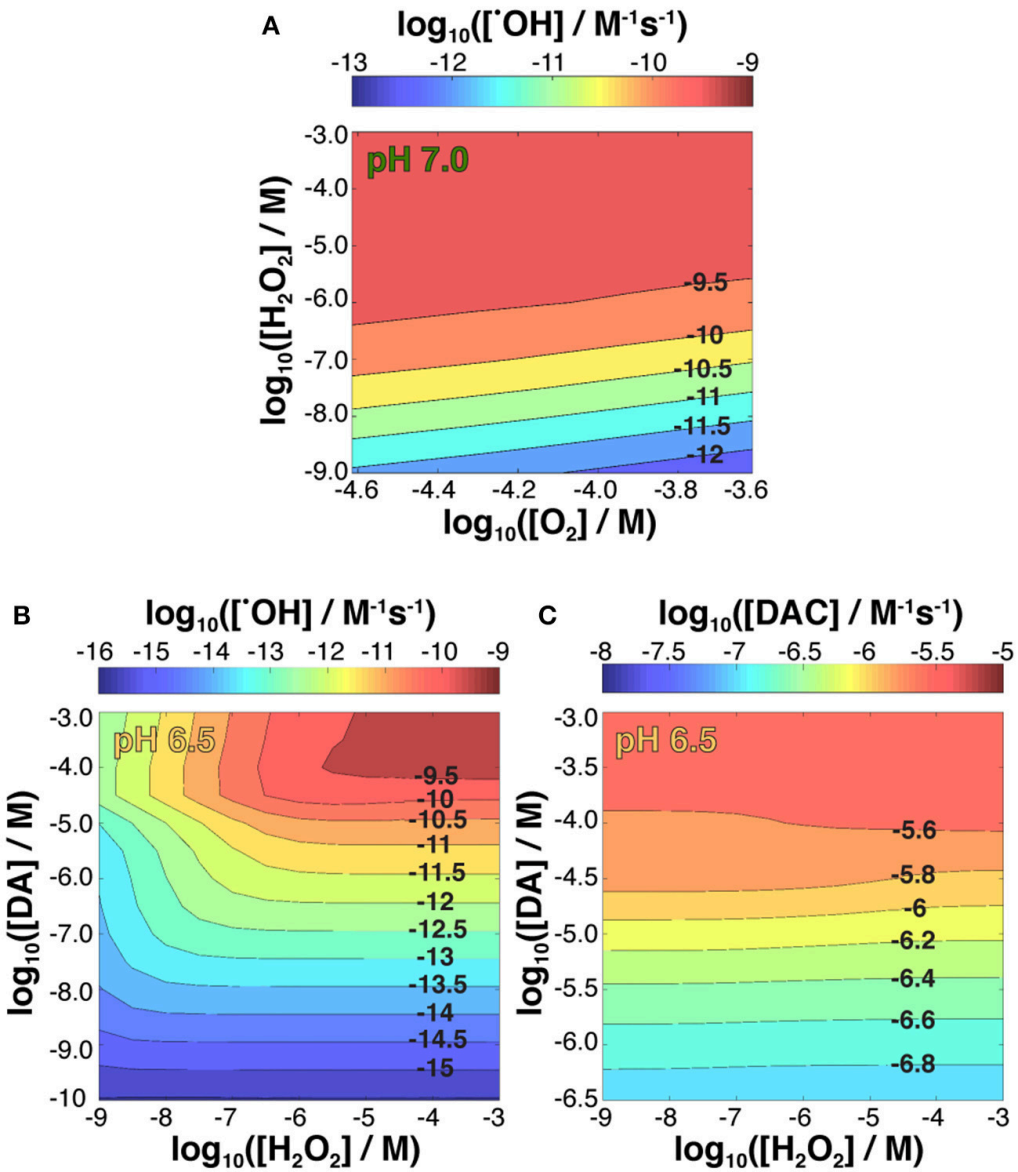
Kinetic modeling of ^•^OH production variability in the presence of 5 μM Fe[II] as a function of both fixed concentrations of O_2_ and H_2_O_2_ and steady-state 50 μM DA at pH 7.0 **(A)**; and ^•^OH **(B)** and DAC **(C)** production as a function of both fixed concentrations of H_2_O_2_ and DA at pH 6.5.

As shown in Figure 6A, at a fixed concentration of 50 μM DA, increased ^•^OH production rate was observed with increasing H_2_O_2_ concentration and decreasing O_2_ concentrations over a 10–60 μM range typical of that within the brain (Ndubuizu and Lamanna, 2007). The increased production of hydroxyl radicals as H_2_O_2_ becomes more concentrated can be attributed to the relatively slow oxygenation rates at these conditions and the potential for active peroxidation of any Fe[II] present. In contrast, the effect of DA concentration on ^•^OH production as predicted by the model was more complex (Figure 6B; Supplementary Figure 10) as DA can both sequester Fe[II] and Fe[III] in a variety of coordination complexes and mobilize Fe[III] from ferric oxide precipitates *via* DA induced dissolution. As a whole, DA concentrations under 0.1 μM produce minimal amounts of ^•^OH regardless of the steady-state H_2_O_2_ concentration with this effect principally a result of there being insufficient labile Fe present to promote oxidation. In contrast, as DA concentrations exceed 10 μM, production of ^•^OH is accelerated, provided the steady-state H_2_O_2_ concentration is in excess of ~100 nM, indicating that the leakage of DA at the later stage may aggravate the Fe and DA induced oxidative stress.

Again, the effect of increasing pH that promotes Fe^III^DA_2_ complex formation partially attenuates ^•^OH production (Supplementary Figure 10), whereas Fe^III^DA production favored at pH 6.5 results in an increase in ^•^OH generation rate (Figure 6B; Supplementary Table 2) compared to the behavior observed at both higher pH values and equivalent DA and H_2_O_2_ concentrations in an Fe-free environment.

Dopamine concentration had the largest effect on DAC production for all pH values investigated (Figure 6C; Supplementary Figure 11) with the highest rate of quinone formation observed at pH 6.5. On the other hand, while the model indicated H_2_O_2_ concentration had minimal influence on the rate of DAC formation at pH 6.5, low concentrations of H_2_O_2_ at pH 7.0 and pH 7.4 favor the accumulation of DAC, particularly at the lower range of DA concentrations used. Highlighting the versatility of Fe redox activity and why it is tightly regulated under normal physiological conditions, these results suggest that the oxidative load in a dopaminergic neuron arising from Fe-mediated ^•^OH production at basal H_2_O_2_ levels are somewhat mitigated by the concomitant oxidation of Fe[II], effectively sequestering Fe in the ferric state, which is less active with respect to neurotoxic DAC production.

## Discussion

The value of this study is in the detailed kinetic and thermodynamic profiling of DA oxidation, which provides direct chemical evidence to support the involvement of this unique source of oxidative stress in a potentially pathogenic mechanism of dopaminergic neurotoxicity.

### Iron, dopamine, and selective neuronal loss in parkinson's disease

Iron accumulates in multiple regions of the aging human brain (Acosta-Cabronero et al., 2016), and is most marked in the Fe-rich deep gray matter within the basal ganglia (Li et al., 2014). The degree of accumulation in Parkinson's disease patients is markedly higher in the SNc and putamen (Wang et al., 2016), where dopaminergic neurons project, and is associated with disease severity (Wallis et al., 2008), suggesting elevated levels of Fe are intrinsically linked to the biochemical mechanism underlying neuron loss (Hare et al., 2015). The disease-specific hyperaccumulation of Fe throughout the nigrostriatal pathway (He et al., 2015) indicates a particular vulnerability of dopaminergic neurons, and a lack of apparent pathology in non-Parkinson's-affected regions that also accumulate Fe during normal aging (Acosta-Cabronero et al., 2016) support the hypothesis that DA oxidation and free radical generation, occurring either independently of, or concurrent to, classical Fenton chemistry is a major source of oxidative stress in Parkinson's disease.

This characteristic feature may explain how two essential neurochemicals, which are normally segregated by vesicular confinement of DA to prevent oxidation by cytoplasmic Fe, are able to interact in the Parkinson's disease brain, and how they relate to α-synuclein dysfunction. Both mutations to the *SNCA* gene encoding α-synuclein and post-translational oxidative damage to the protein (Lotharius and Brundin, 2002b), the latter being an effect of elevated levels of Fe and Fenton-type ROS production, can lead to permeabilized vesicles that effectively “leak” DA into the pro-oxidant environment of the cytoplasm (Lotharius and Brundin, 2002a). This initial ^•^OH-driven oxidative damage may trigger a cascade of free radical production that accelerates the rate of α-synuclein modifications, impaired vesicular transport, and DA oxidation, in turn, aggravating cell loss by triggering DAC-induced neurotoxicity.

Central to the view that elevated Fe facilitates DA breakdown is the notion of a labile Fe pool. The propensity of unbound cytoplasmic Fe[II] to react with by-products (such as H_2_O_2_) of mitochondrial respiration necessitates tight metabolic regulation of neuronal Fe levels involving various regulatory proteins (Moos et al., 2007), a number of which have been recognized as being dysfunctional in Parkinson's disease (Hare et al., 2013). Whether a labile Fe pool exists in actuality is the subject of much debate and both rapid oxidation of Fe[II] in the O_2_-rich environment and obvious difficulties in directly speciating Fe within a living human neuron represent two analytical challenges that preclude obtaining conclusive evidence that Fe dyshomeostasis actively promotes increased oxidative stress in Parkinson's disease. Regardless, as nigral Fe accumulation is now recognized as an indisputable pathological feature of Parkinson's disease (Ayton and Lei, 2014; Wang et al., 2016), understanding the mechanism of how Fe promotes oxidative stress in dopaminergic neurons is critical as Fe chelation therapies enter clinical trials (Devos et al., 2014; Martin-Bastida et al., 2017; Moreau et al., 2018; Sun et al., 2018c).

### Iron-induced dopamine radical production is independent of fenton chemistry

Iron-mediated DA oxidation differs from Fenton/Haber-Weiss chemistry in that, in addition to redox cycling of Fe[II]/Fe[III], both species are capable of forming intermediary complexes immediately preceding free radical production. For instance, both Fe[II] and Fe[III] are capable of *d* orbital “bridging” between DA and O_2_, and inducing the subsequent accelerated transfer of electrons and generation of DA^•−^ and O${}_{2}^{•-}$ free radicals (Miller et al., 1990). The reactions and associated rate constants involved in Fe[II]/Fe[III] redox cycling and DA oxidation are listed in Tables 2, 3.

The particularly strong ability of Fe[II] to generate DAC and H_2_O_2_ evident in Figures 2, 3 may result from the relative insolubility and precipitation of Fe[III] as AFO over the pH range investigated. This is an important factor to consider, as ferritin sequesters Fe within a protein nanocage in a chemical state resembling AFO (Jian et al., 2016). However, the ready oxidation of Fe[II] in the presence of DA (Reaction 24 and Reaction 25 in Table 3) favors the conversion of Fe[II] to the Fe^[III]^DA complex and subsequent O${}_{2}^{•-}$ generation (Sun et al., 2016). Fe^[III]^DA is not particularly stable and may either react with another DA molecule to form Fe^III^DA_2_ (shown in Figure 4C and Supplementary Figure 7C), reversibly react with O${}_{2}^{•-}$ to reform Fe^[II]^DA and O_2_ or undergo ligand-to-metal charge transfer (LMCT) with release of Fe[II] and DA^•−^ as follows:

$$\begin{array}{r} {\text{Fe}}^{\left[\text{III}\right]}\text{DA}\to \text{Fe}\left[\text{II}\right]+{\text{DA}}^{•-} \end{array}$$

Compared to the relatively inactive iron oxyhydroxide precipitates, the more soluble DA-bound Fe[III] oxidation product formed from ferrous DA complexes should enhance the production of both DAC and H_2_O_2_ by accelerating generation of both DA^•−^ and O${}_{2}^{•-}$ radicals.

Distinguishing iron-mediated dopamine oxidation from Fenton-generated ROS is important, as the free radicals produced do not have the multiple redundant detoxification pathways that exist for H_2_O_2_. While quinones are a relatively minor component of dopamine metabolism, which ordinarily favors providing a precursor for adrenaline biosynthesis or excretion from the central nervous system as homovanillic acid, it is unclear precisely how these highly-reactive species are neutralized. Several endogenous enzymes have been shown to interact with dopamine *o*-quinones, including SOD1, glutathione transferase (by way of glutathione conjugation) and macrophage migration inhibitory factor (Solano et al., 1999; Haque et al., 2003), though quinone detoxification would be considered a secondary function. In the absence of metal catalysis, polymerization of quinones to neuromelanin is a remarkably slow process with a normal aged brain containing only around 5 mg g^−1^ (Aime et al., 2000). Without an efficient mechanism of quinone removal, these products have greater neurotoxic potential. This is especially the case in human dopaminergic neurons, in light of recent data indicating that dopaminergic neurons derived from fibroblasts of sporadic and familial Parkinson's disease patients contain higher concentrations of DA and oxidation products than similarly-prepared murine cell cultures (Burbulla et al., 2017).

### Relevance of iron and pH to disease progression

The rapid formation of both DAC and H_2_O_2_ may be attributed to the pH-dependent oxidation of DA-bound Fe[II] shown in Figure 4A. Variation in pH reflecting transition from a physiologically normal microchemical environment to Parkin-associated mitochondrial dysfunction (Pickrell and Youle, 2015) and *post-mortem* acidosis (Donaldson and Lamont, 2013) had a marked effect on DAC and H_2_O_2_ production concomitant to pH-dependent oxidation of Fe[II]. The catechol moiety of DA is capable of forming a strong complex with Fe[III] (Avdeef et al., 1978; Sever and Wilker, 2004):

$$\begin{array}{r} \text{Fe}\left[\text{III}\right]+\text{DA}\to {\text{Fe}}^{\left[\text{III}\right]}\text{DA} \end{array}$$

with the *mono*-complex (Fe^[III]^DA) dominant at acidic pH and the *bis* Fe^[III]^DA_2_ complex favored at circumneutral pH (Supplemental Figure 4). At pH 6.5, the ratio of [Fe^[III]^DA_2_]/[Fe^[III]^DA] is ~5 in conditions reflective of otherwise physiologically normal concentrations of Fe and DA. Once formed, radical generation would also occur, increasing potential protein and lipid peroxidation events with the rate of LMCT for Fe^[III]^DA (0.23 s^−1^) substantially higher than that for Fe^[III]^DA_2_ (7.26 × 10^−5^ s^−1^; El-Avaan et al., 1997; Sun et al., 2016). From the rate law

$$\begin{array}{r} \frac{d\;\left[\text{Fe}\left[\text{II}\right]\right]}{dt}\;=\;\frac{d\;\left[\text{D}{\text{A}}^{•-}\right]}{dt}\;=\;k\left[\text{F}{\text{e}}^{\left[\text{III}\right]}\text{DA}\right] \end{array}$$

continuous generation of DAC at pH 6.5, especially in the presence of Fe[II], was unsurprising. The slightly higher concentrations of DAC formed in the presence of Fe[III], particularly during the initial half of the total reaction time assessed (Figure 2C), may be attributed to the more efficient Fe mobilization induced by DA at higher pH.

In contrast to the slower yet continuous generation of H_2_O_2_ at pH 7.0 and 7.4 (Figure 3A), the non-linear formation of DAC at these pHs (Figure 2B) suggests that DAC levels are maintained at a steady-state under physiological conditions through rearrangement of DAC and the subsequent formation of indoles or even possibly neuromelanin (Supplementary Figure 8). It is reasonable to deduce that the rearrangement of DAC coupled with the ensuing polymerization on increase in pH plays an important role in the removal of DAC.

It should be noted that the presence of metals, such as Fe and calcium (as Ca^2+^) can also promote this process (Sun et al., 2018a). The model developed adequately describes the generation of reactive oxygen species and accumulation of DAC but comprehensive description of the specific role of Fe in DAC decomposition will require an expanded model. Regardless, the accumulation of protein-modifying DAC is likely to be enhanced at low pHs as a result of the presence of high concentrations of the active Fe[II] catalyst seen in both parkinsonian animal models (Hare et al., 2014; Billings et al., 2016) and the human Parkinson's disease brain (Dexter et al., 1991).

The cessation of H_2_O_2_ accumulation observed at pH 6.5 (Figure 3A) may be related to the high concentration of Fe[II] present in the solution. Under these conditions, the H_2_O_2_ initially generated as a result of DA-enhanced oxidation of Fe[II] is subsequently consumed *via* peroxidation of the remaining Fe[II]. Apart from the obvious pH-dependent oxidation process, attenuation of Fe[II] oxidation at pH 6.5 (Figure 4A), especially during the later stages of the reaction, suggests that Fe[II] may be regenerated under these conditions with the regeneration of Fe[II] related to formation of DA intermediates such as DA^•−^. Given the continuous generation of DAC (Figure 2B), the high concentrations of Fe[II] observed may, in turn, result from DA^•−^-induced reduction of ferric iron. Thermodynamically, the low reduction potential of the DAQ/DA^•−^ couple indicates that reduction of aqueous Fe[III] is feasible (Pham and Waite, 2014). Indeed, the rapid reduction of Fe[III] by 6-hydroxydopamine (6-OHDA) semiquinones has been proposed (Jameson and Linert, 2001) with 6-OHDA itself a neurotoxic breakdown product of DA that is facilitated by Fe (Hare and Double, 2016). However, as pH increases, it is expected that the majority of the Fe present would be efficiently converted into AFO and, with a low reduction potential at pH 7.0 (Watt et al., 1985), DA^•−^ is unable to mobilize Fe from AFO. It is of note that neuronal ferritin iron is stored in an AFO-like ferrihydrite core (Hagen et al., 2017) which is prone to mobilization under acidic conditions (La et al., 2017).

### Considerations for analysis of dopamine metabolites in *post-mortem* tissue

When viewed within the context of existing literature, the results described here present something of a conundrum for those wishing to quantify DAC and related DA oxidation products in human SNc tissue. While acidosis occurring in degenerating neurons may contribute to the progression of Parkinson's disease by increasing the rate of oxidant generation and accumulation of DAC, *post-mortem* decreases in tissue pH, stemming from sample handling, exposure to the environment after removal at autopsy, and even *post-mortem* interval may present inaccurate assessment of DA oxidation.

Considering mitochondrial dysfunction is common to most neurodegenerative diseases (Lin and Beal, 2006), it is not surprising that data supplied from tissue housed in the Sydney Brain Bank, where 72% of cases with a neurodegenerative disease, including pathologically-diagnosed Parkinson's disease, exhibited tissue pH as low as 5.86 (Genoud et al., 2017). Tissue pH that deviates from normal physiology, regardless of whether the cause is endogenous or artifact, will undoubtedly alter the speciation of DA and Fe, and rate of formation, reactivity and fate of toxic DA intermediates (El-Avaan et al., 1997; Pham et al., 2006; Sun et al., 2015, 2016, 2018a). Accordingly, to avoid any misinterpretation of DA metabolites, quantitative assessment of DA metabolism in *post-mortem* tissue should account for multiple confounding factors that influence tissue pH.

## Conclusions

**Figure 7:**
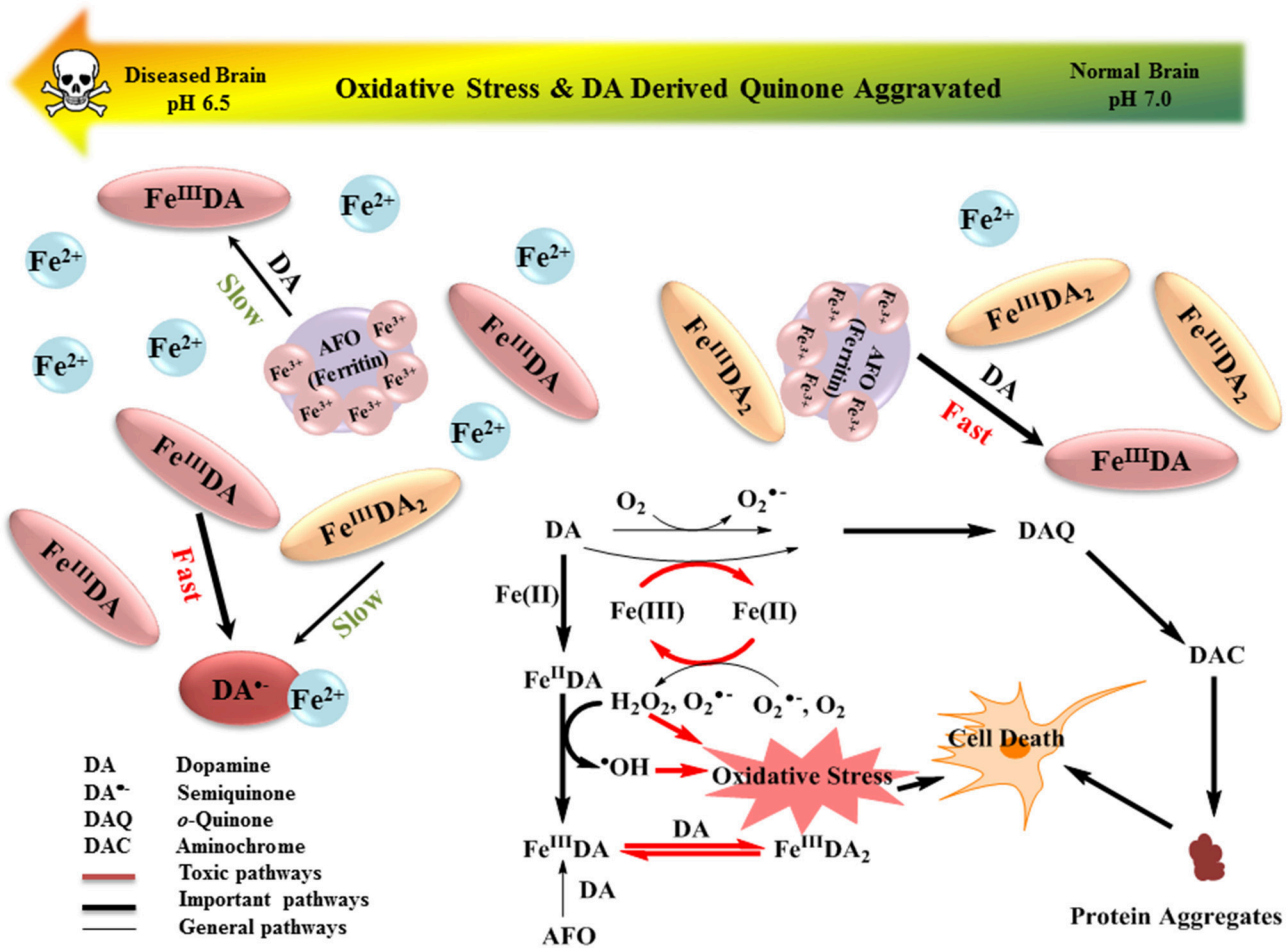
Schematic showing the relative importance of the various reactions involved in the interplay between DA and Fe. Expected changes in key metabolites on decrease in pH are also shown.

The results of this study indicate that the formation of DA bound Fe[II] and Fe[III] complexes as well as the cyclization and rearrangement of DA-derived quinones are the most important pathways of DA metabolism, with each process heavily dependent on pH. A schematic showing the relative importance of these processes together with expected changes in key metabolites on decrease in pH is presented in Figure 7. The presence of Fe can accelerate the oxidation of DA and the accumulation of deleterious protein-modifying DAC with Fe[II] being more efficient in this regard than Fe[III]. Although DAC was slowly formed at pH 6.5, the decrease in the rearrangement rate of this species in acidic conditions resulted in long-term accumulation of DAC. As a result of both the rapid reduction of the unbound-Fe[III] by DA^•−^ and the enhanced LMCT that occurs with the change in the speciation of DA-bound Fe, a substantially higher concentration of Fe[II] was generated at pH 6.5 compared to that at pH 7.0 and 7.4. Even though the presence of both Fe[III] and Fe[II] resulted in increased accumulation of H_2_O_2_, the enhanced Fe[II] regeneration coupled with the slow rate of oxygenation of Fe[II] at pH 6.5 gave rise to substantially greater potential for the peroxidation of Fe[II] with concomitant enhanced generation of ^•^OH. Model predictions indicate that, in the presence of the same concentrations of DA and H_2_O_2_, acidosis in the Parkinson's disease brain results in an increase in DAC accumulation and DA-mediated production of hydroxyl radicals, with these potential toxicants likely to further aggravate the progression of Parkinson's disease and severity of symptoms arising from DA denervation.

The model developed in this study places emphasis on the purely chemical interactions between Fe and DA and is of particular value in facilitating prediction of the long-term consequences of these interactions by incorporating a range of conditions resulting from the complicated *in vivo* homeostasis of dopaminergic neurons such as continuous DA leakage into the cytosol and increased steady-state concentrations of H_2_O_2_ as a result of the dysfunctional of antioxidant enzymes. It is also important to view these data within the broader biochemical context of Parkinson's disease neuropathology as a result of the limitations of current techniques. Regardless, the data presented here shows that pH, Fe and H_2_O_2_ concentrations are intrinsically linked in the rate of formation of neurotoxic DA metabolites.

## Author contributions

All the authors were involved in the experiments design. YS conducted the experiments and prepared the manuscript. AP, DH, and TW revised the manuscript. All authors reviewed the results and approved the final version of the manuscript.

### Conflict of interest statement

DH receives research and materials support from Agilent Technologies. The remaining authors declare that the research was conducted in the absence of any commercial or financial relationships that could be construed as a potential conflict of interest.

## Abbreviations

6-OHDA: 6-hydroxydopamine
AFO: amorphous iron oxide
DA: dopamine (1-amino-2-(3,4-dihydroxyphenyl)ethane)
DA^•−^: semiquinone radical
DAC: dopaminochrome
DAL: leukoaminochrome
DAQ: dopamine-*o*-quinone
DHI: 5,6-dihydroxyindole
ECF: extracellular fluid
Fe[II]: inorganic ferrous ion
Fe[III]: inorganic ferric ion
Fe^[III]^DA: *mono*-complex
Fe^[III]^DA_2_: *bis*-complex
Fe^[III]^DA_3_: *tris*-complex
Fe[III]_I_: total inorganic Fe[III]
H_2_O_2_: hydrogen peroxide
LMCT: ligand to metal charge transfer
MQ: Milli-Q water
O${}_{2}^{•-}$: superoxide
^•^OH: hydroxyl radical
ROS: reactive oxygen species
SNc: substantia nigra pars compacta
TOR: turnover rate.
