# Supplementary material for: Kinetic Modeling of pH-Dependent Oxidation of Dopamine by Iron and Its Relevance to Parkinson's Disease
Source: Front Neurosci. 2018 Nov 26;12:859. doi: 10.3389/fnins.2018.00859 (PMC6275323; doi:10.3389/fnins.2018.00859)
Supplement: Supplementary file 1 [file Data_Sheet_1.pdf]

Supplementary Material

Kinetic modelling of pH-dependent oxidation of dopamine by iron and its relevance to Parkinson's disease

Yingying Sun, A. Ninh Pham, Dominic J. Hare and T. David Waite*

*** Correspondence:** Corresponding Author: Professor T. David Waite, Tel.: +61 2 9385 5059, Email: d.waite@unsw.edu.au

1 Supplementary Materials and methods

Preparation of analytical reagents

A concentrated Fe[II] stock solution (5 mM) was prepared by dissolving ferrous ammonium sulfate hexahydrate ($Fe(NH_4SO_4)_2 \cdot 6H_2O$) in 10 mM HCl. Concentrated stock solutions of 10 mM Fe[III] as ferric chloride hexahydrate ($FeCl_3 \cdot 6H_2O$) and 10 mM DA were prepared weekly in 10 mM HCl. The working stock solutions of Fe[II], Fe[III] and DA were diluted from the concentrated stock solutions daily in 10 mM HCl. The low pH of both concentrated stock and working stock solutions was sufficient to prevent significant oxidation of Fe[II] and DA and precipitation of Fe[III] during the course of each experiment while also minimizing pH change following addition of the stocks to experimental solutions. A stock solution of 20 mM H_2O_2 prepared by dilution of a nominal 30% H_2O_2 (w/w) solution was used to calibrate H_2O_2 measurements. The 30% H_2O_2 solution was standardized using an Agilent Technologies Cary 60 UV-Vis spectrophotometer (Mulgrave, Australia) at 240 nm, which was subsequently used for all spectrophotometric measurements.

Concentrated stock solutions of 80 mM FZ and 20 mM desferrioxamine B (DFO-B; *N*'-[5-(acetyl-hydroxy-amino)pentyl]-*N*-[5-[3-(5-aminopentyl-hydroxycarbamoyl)propanoylamino] pentyl]-*N*-hydroxy-butane diamide) were prepared in MQ. A mixture containing 50 mM FZ and 5 mM DFO-B was prepared daily and used for Fe[II] determination. Stock solutions of 6 mM *N,N*-diethyl-*p*-phenylenediamine (DPD) and 500 kU L^{-1} horseradish peroxidase (HRP) were prepared in MQ as described previously (Bader et al., 1988). A 10 mM stock solution of diethylenetriaminepentaacetic acid (DTPA) was prepared in 10 mM MOPS and the pH adjusted to 6.0. A stock solution of 20 mM 2,2′-bipyridyl (BPY) was prepared in 5 mM HCl. A 20 mM $NaIO_4$ stock solution was prepared monthly in MQ. A 1 mM DAC working solution was prepared by adding 1 mM DA and 2 mM $NaIO_4$ into 20 mM HCl immediately prior to each set of experiments (Sun et al., 2018a).

2 Supplementary Figures

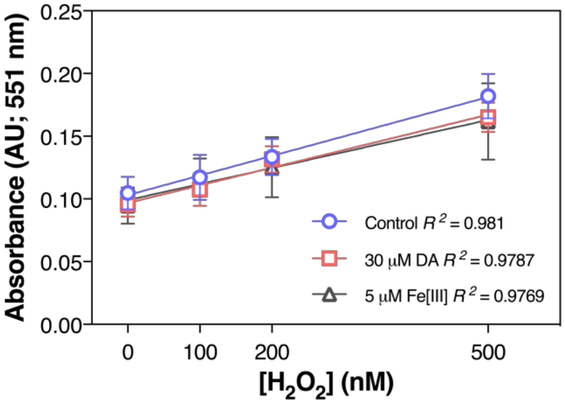

Supplementary Figure 1. Measured absorbance and linear regression of H_2O_2 standards at 551 nm, baseline corrected at 690 nm, in the pH 6.5 measurement buffer (blue circles). Addition of 30 μM DA (red squares) or 5 μM Fe[II] (grey triangles) did not affect H_2O_2 absorbance measurements. Error bars are standard errors from duplicate measurements.

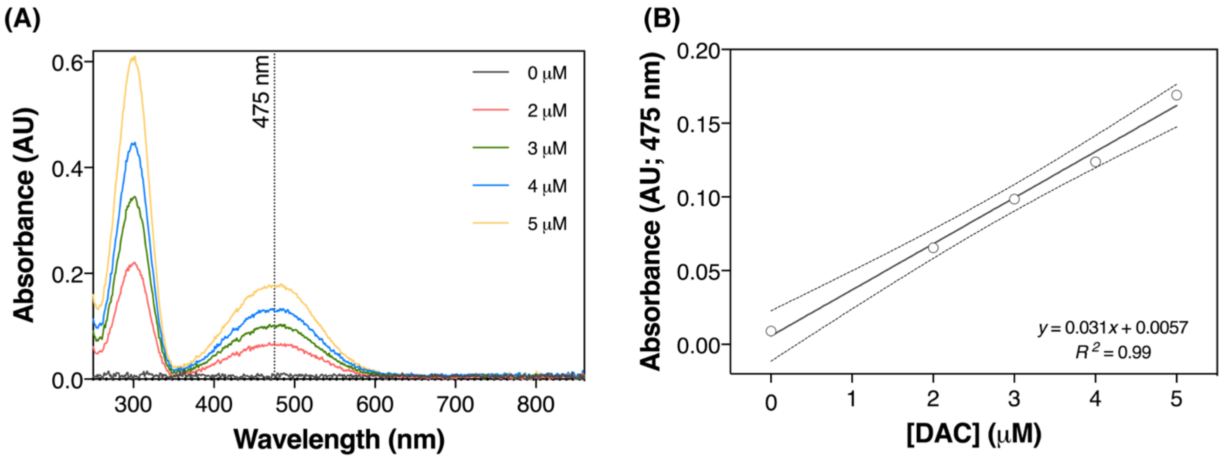

Supplementary Figure 2. Measured absorbance of DAC (A) and calibration curve for qualification of DAC measured at 475 nm with baseline correction at 850 nm (B) in 0.1 M NaCl. Dashed line = 95% confidence interval.

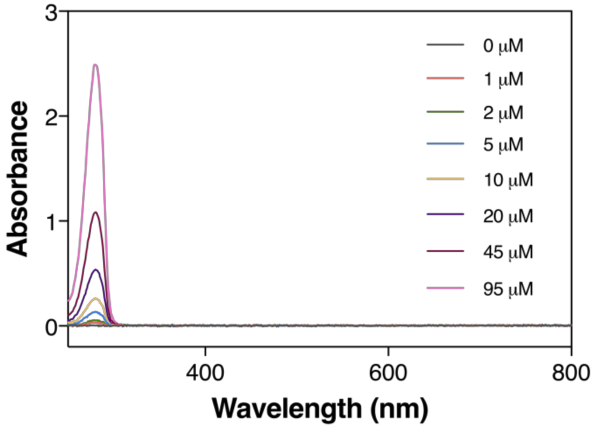

Supplementary Figure 3. Measured absorbance of DA in 0.1 M NaCl.

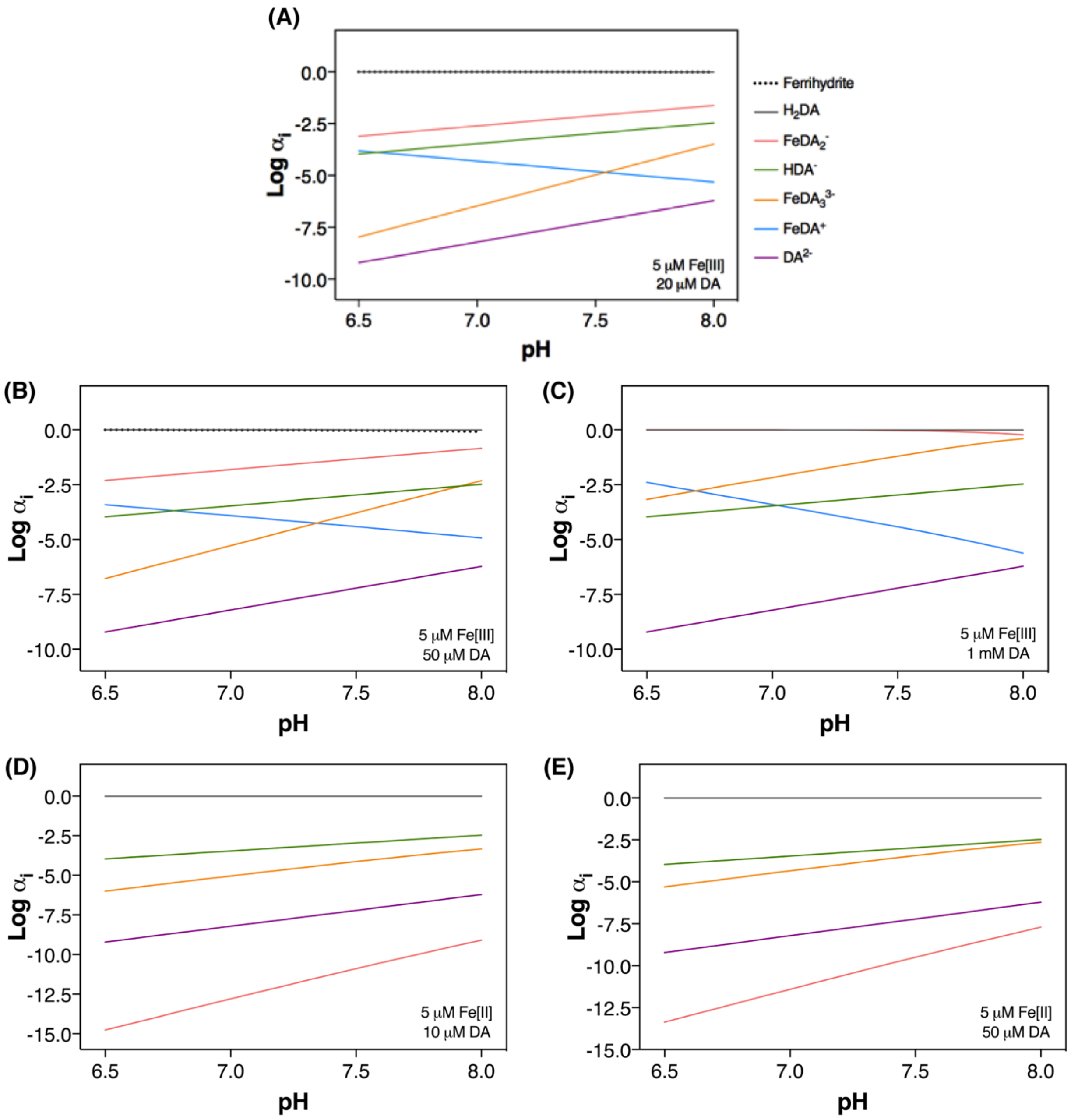

Supplementary Figure 4. Distribution of different species of Fe[III] (A, B and C) and Fe[II] (D and E) over the pH range 6.5 - 8.0 in solutions containing varying concentrations of DA with $[Fe[III]]_0$ = 5 μM or $[Fe[II]]_0$ = 5 μM. α_i denotes the molar fraction of individual species.

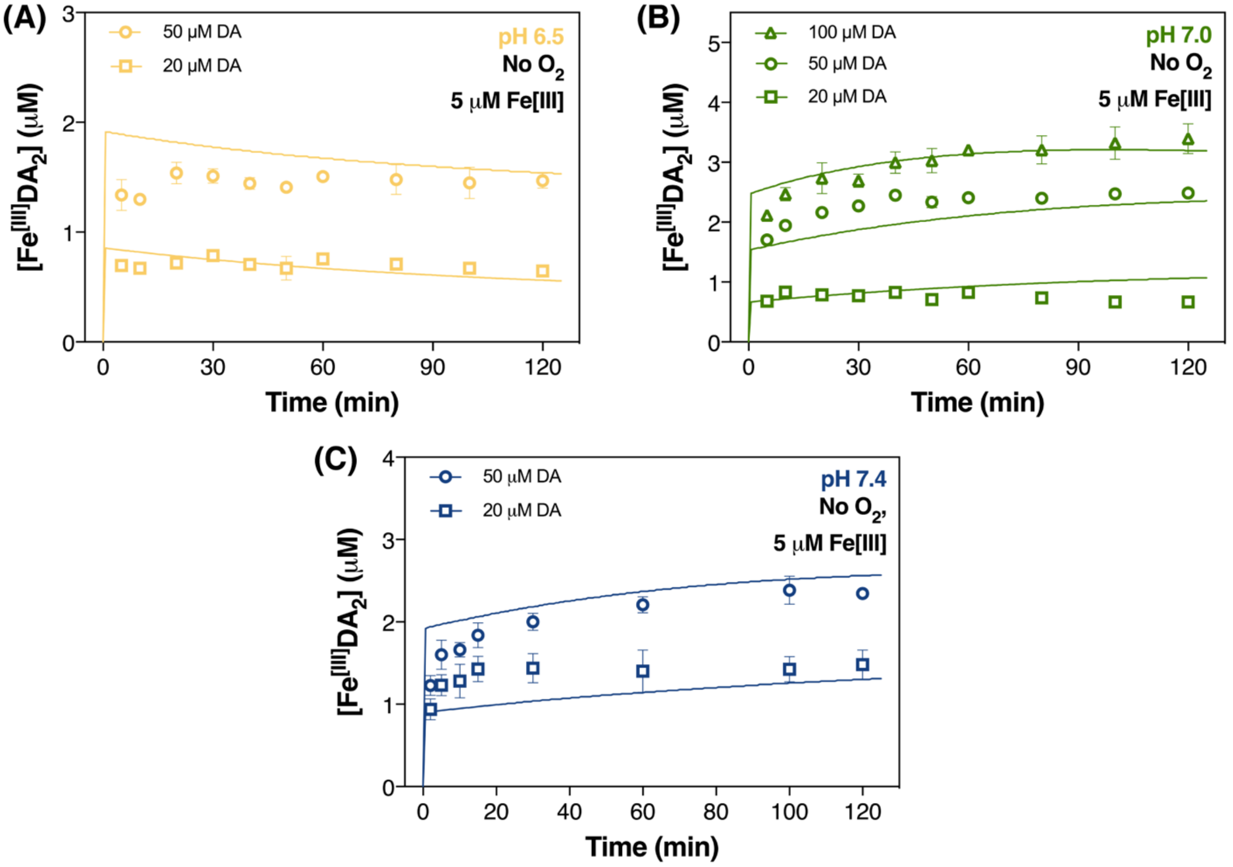

Supplementary Figure 5. Formation of $Fe^{[III]}DA_2$ followed by the addition of 5 μM Fe[III] to 0.1 M deoxygenated NaCl solutions containing 20 μM DA or 50 μM DA, at pH 6.5 (A), pH 7.0 (B, plus 100 μM DA) and pH 7.4 (C). Error bars are standard errors from triplicate measurements and solid lines represent the model fit. The data used at pH 7.4 was taken from Sun et al. (2016) but the solid lines represent the model modified in this study.

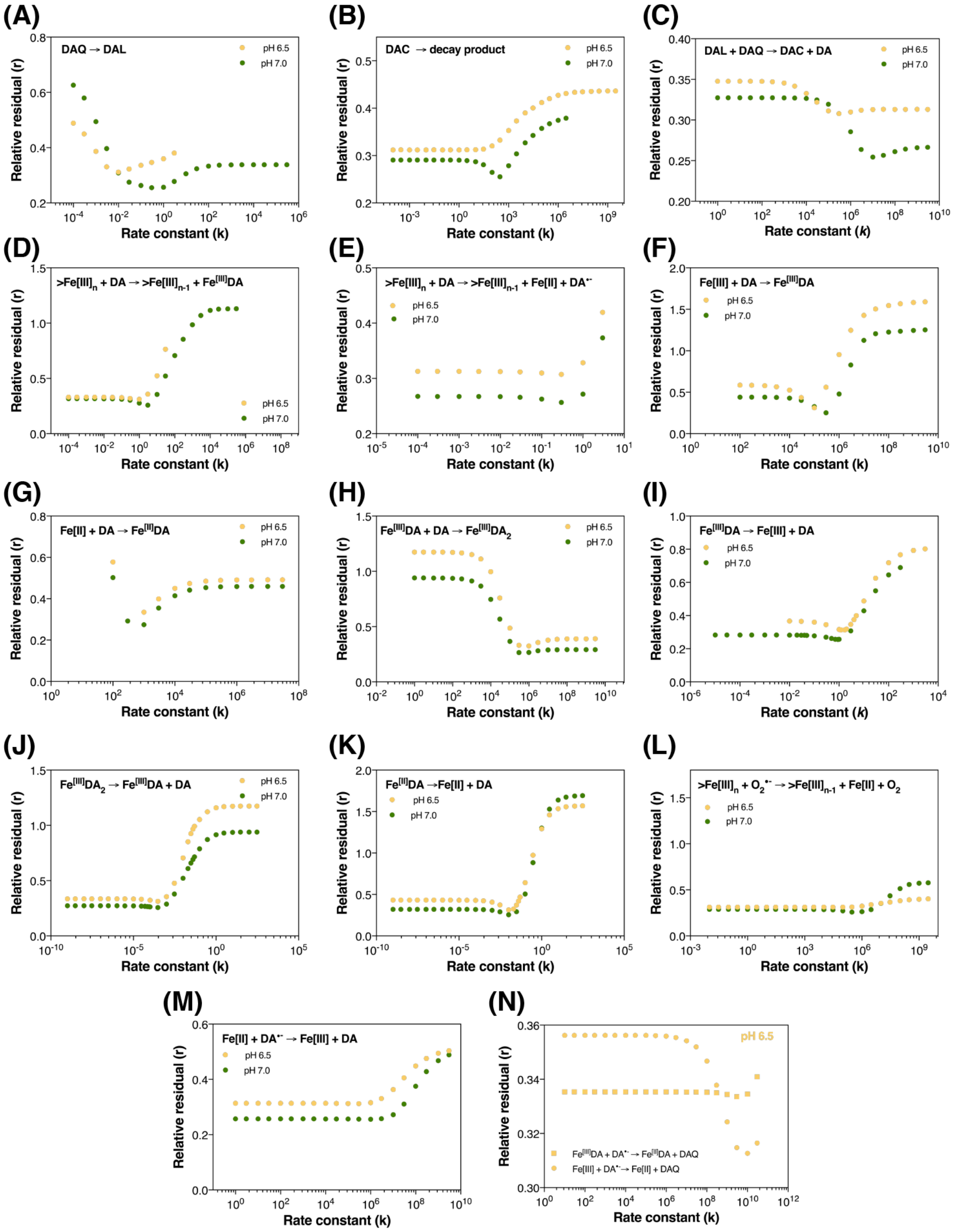

Supplementary Figure 6. Sensitivity analysis for the fitted rate constants of different reactions (**Tables 1-3**, main text).

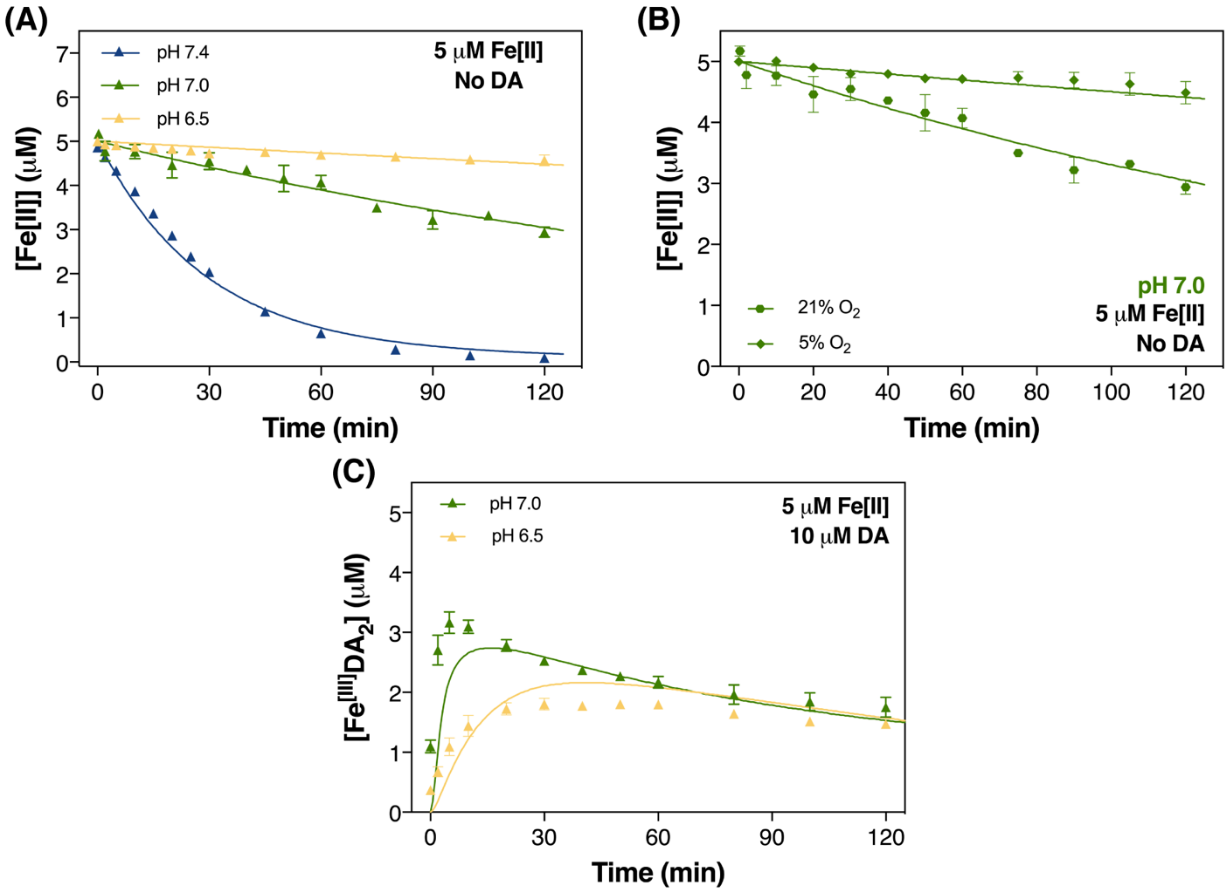

Supplementary Figure 7. Oxidation of 5 μM Fe[II] in the absence of DA at pH 6.5, pH 7.0, and pH 7.4 in 0.1 M NaCl (A); in the absence of DA at pH 7.0 in 0.1 M NaCl solutions containing 5% O_2 and 21% O_2 (B); and formation of $Fe^{[III]}DA_2$ in the presence of 5 μM Fe[II] and 10 μM DA at pH 6.5 and pH 7.0 in 0.1 M NaCl (C). Error bars are standard errors from triplicate measurements and solid lines represent the model fit. The data used at pH 7.4 was taken from Sun et al. (2016) and the solid lines represent the model modified in this study.

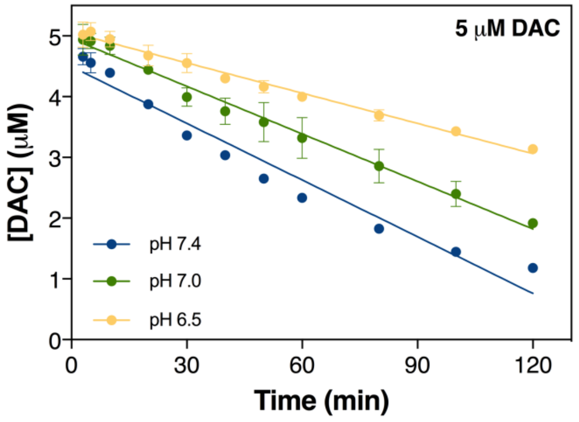

Supplementary Figure 8. Decay of 5 μM DAC in the absence of added iron at pH 6.5, pH 7.0, and pH 7.4 in 0.1 M NaCl.

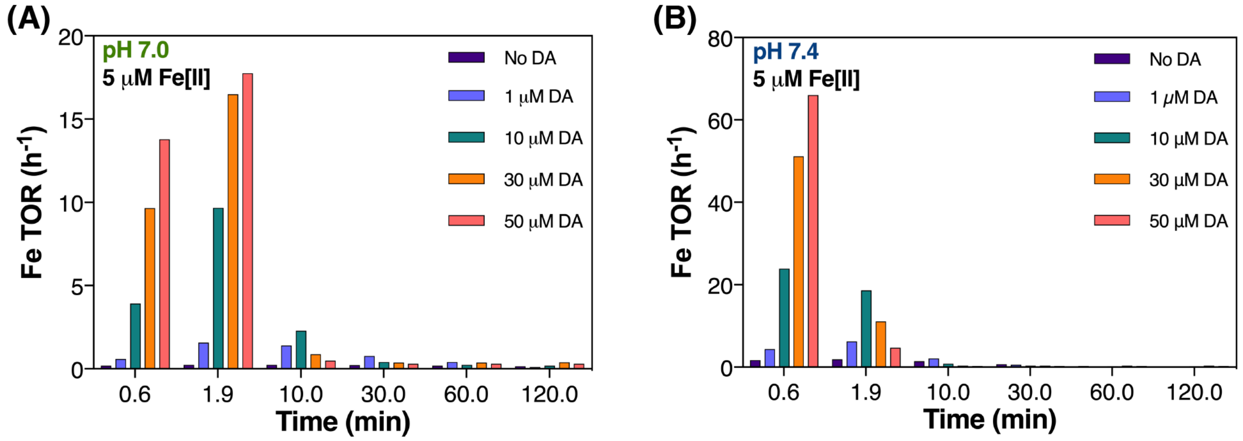

Supplementary Figure 9. Turnover rate of Fe[II] in air-saturated (21% O_2) 0.1 M NaCl solutions in the presence of 0-50 μM DA at pH 6.5 (A) and pH 7.4 (B).

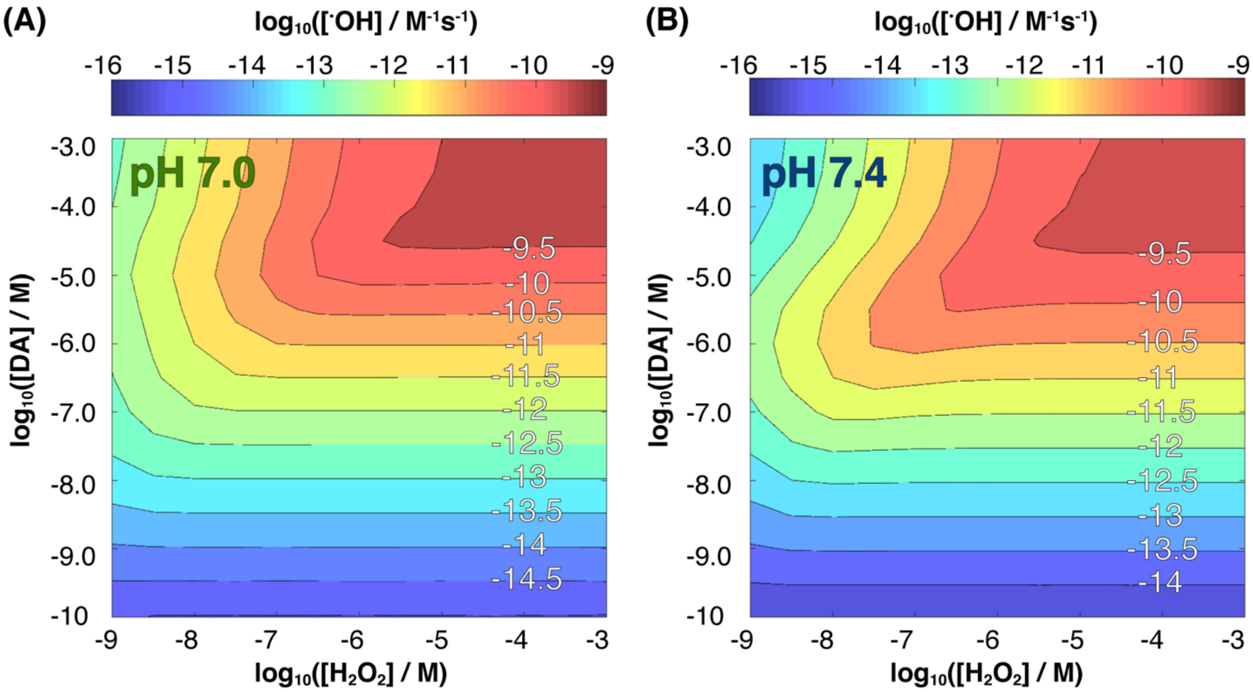

Supplementary Figure 10. Variability of the production rate of $^{\bullet}OH$ as a function of fixed H_2O_2 and DA concentrations at pH 7.0 (A) and pH 7.4 (B).

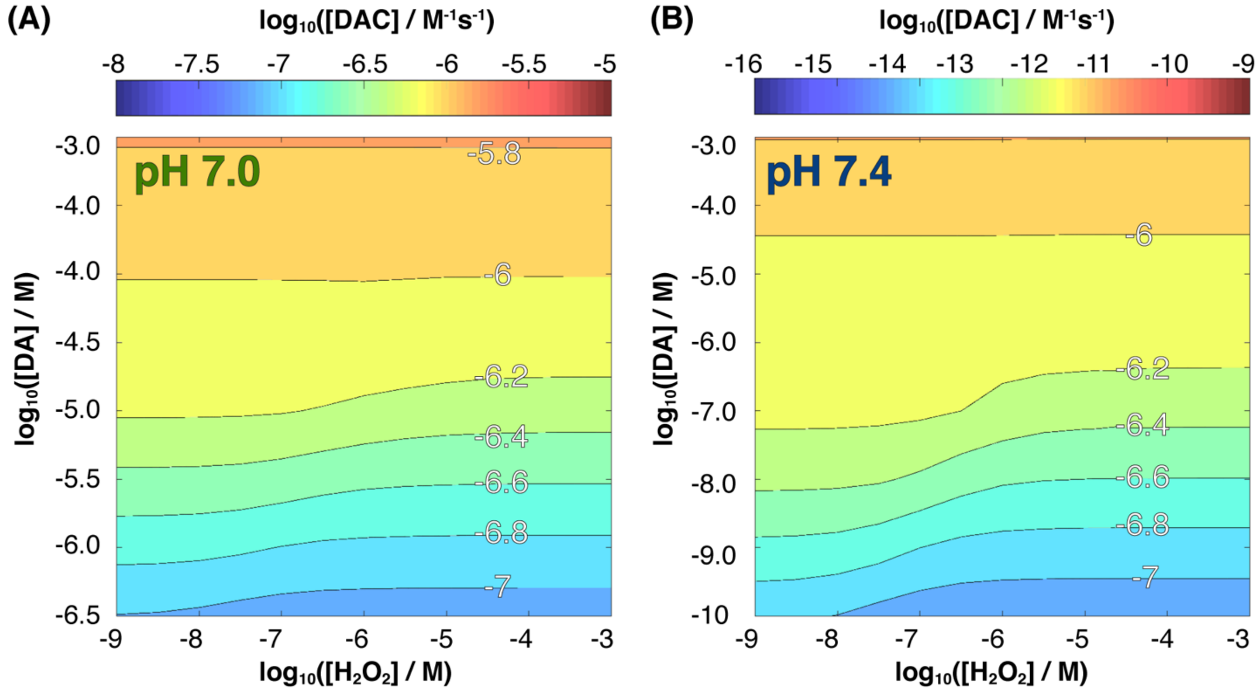

Supplementary Figure 11. Variability of steady-state DAC concentration as a function of fixed H_2O_2 and DA concentrations at pH 7.0 (A) and pH 7.4 (B).

3 Supplementary Tables

Supplementary Table 1. Stability constants for Fe(II) and Fe(III) speciations at 25 °C and $I = 0$.

No.	species	Log*K*	Reference
Fe(II) species			
1	$Fe^{2+} + H_2O \rightleftharpoons FeOH^+ + H^+$	-9.51	1
2	$Fe^{2+} + 2H_2O \rightleftharpoons Fe(OH)_2^0 + 2H^+$	-20.6	1
3	$Fe^{2+} + CO_3^{2-} \rightleftharpoons FeCO_3^0$	5.69	2
4	$Fe^{2+} + H^+ + CO_3^{2-} \rightleftharpoons FeHCO_3^+$	11.8	3
5	$Fe^{2+} + 2CO_3^{2-} \rightleftharpoons Fe(CO_3)_2^{2-}$	7.45	2
6	$Fe^{2+} + CO_3^{2-} + H_2O \rightleftharpoons Fe(OH)CO_3^- + H^+$	-4.03	2
7	$Fe^{2+} + Cl^- \rightleftharpoons FeCl^+$	0.3	2
8	$Fe^{2+} + SO_4^{2-} \rightleftharpoons FeSO_4^0$	2.42	2
9	$Fe^{2+} + DA^{2-} \rightleftharpoons FeDA^0$	9.12	4
10	$Fe^{2+} + 2DA^{2-} \rightleftharpoons FeDA_2^{2-}$	14.56	4
Fe(III) species			
11	$Fe^{3+} + H_2O \rightleftharpoons Fe(OH)^{2+} + H^+$	-2.13	5
12	$Fe^{3+} + 2H_2O \rightleftharpoons Fe(OH)_2^+ + 2H^+$	-6.13	5
13	$Fe^{3+} + 3H_2O \rightleftharpoons Fe(OH)_3^0 + 3H^+$	-14.3	5
14	$Fe^{3+} + 4H_2O \rightleftharpoons Fe(OH)_4^- + 4H^+$	-22.2	5
15	$Fe^{3+} + Cl^- \rightleftharpoons FeCl^{2+}$	1.28	5
16	$Fe^{3+} + 2Cl^- \rightleftharpoons FeCl_2^+$	1.16	5
17	$Fe^{3+} + SO_4^{2-} \rightleftharpoons Fe(SO_4)^+$	4.27	5
18	$Fe^{3+} + 2SO_4^{2-} \rightleftharpoons Fe(SO_4)_2^-$	6.11	5
19	$Fe^{3+} + 2CO_3^{2-} \rightleftharpoons Fe(CO_3)_2^-$	19.6	5
20	$Fe^{3+} + DA^{2-} \rightleftharpoons FeDA^+$	21.42	6
21	$Fe^{3+} + 2DA^{2-} \rightleftharpoons FeDA_2^-$	36.46	6
22	$Fe^{3+} + 3DA^{2-} \rightleftharpoons FeDA_3^{3-}$	45.08	6
Aqueous species			
23	$H^+ + OH^- \rightleftharpoons H_2O$	14	7
24	$H^+ + CO_3^{2-} \rightleftharpoons HCO_3^-$	10.3	7
25	$2H^+ + CO_3^{2-} \rightleftharpoons H_2CO_3^*$	16.7	7
26	$NH_3 + H^+ \rightleftharpoons NH_4^+$	9.24	1
27	$H^+ + SO_4^{2-} \rightleftharpoons HSO_4^-$	1.99	1

28	$Na^+ + CO_3^{2-} \rightleftharpoons NaCO_3^-$	1.27	2
29	$Na^+ + H^+ + CO_3^{2-} \rightleftharpoons NaHCO_3^0$	10.1	2
30	$Na^+ + SO_4^{2-} \rightleftharpoons NaSO_4^-$	1.06	2
31	$NH_4^+ + SO_4^{2-} \rightleftharpoons NH_4SO_4^-$	1.03	8
32	$H_2DA \rightleftharpoons HDA^- + H^+$	-10.58	9
33	$HDA^- \rightleftharpoons DA^{2-} + H^+$	-12.07	9

(1) Morel and Hering (1993); (2) King (1998); (3) Millero and Hawke (1992); (4) Smith and Martell (1989); (5) Pham et al. (2006); (6) Avdeef et al. (1978); (7) Millero et al. (1995); (8) Schecher and McAvoy (1992) and (9) Pham and Waite (2014).

Supplementary Table 2. Variability of the production rate of $^{\bullet}OH$ as a function of fixed H_2O_2 and DA concentrations at pH 6.5, 7.0 and 7.4

$\log[H_2O_2]$	$\log[DA]$ (M)							
(M)	-10	-9	-8	-7	-6	-5	-4	-3
pH 6.5								
-9	-15.537	-14.690	-14.035	-13.630	-13.463	-12.990	-12.254	-12.420
-8	-15.552	-14.557	-13.606	-12.870	-12.514	-12.002	-11.259	-11.424
-7	-15.548	-14.552	-13.555	-12.595	-11.827	-11.113	-10.304	-10.462
-6	-15.549	-14.550	-13.551	-12.555	-11.602	-10.635	-9.607	-9.727
-5	-15.549	-14.546	-13.551	-12.552	-11.572	-10.572	-9.372	-9.474
-4	-15.551	-14.551	-13.551	-12.552	-11.571	-10.595	-9.263	-9.429
-3	-15.543	-14.553	-13.550	-12.552	-11.571	-10.600	-9.218	-9.393
pH 7.0								
-9	-14.827	-14.060	-13.232	-12.668	-12.423	-12.229	-12.504	-12.654
-8	-15.026	-14.024	-13.026	-12.070	-11.497	-11.238	-11.507	-11.656
-7	-15.026	-14.026	-13.023	-12.017	-10.997	-10.323	-10.534	-10.679
-6	-15.022	-14.024	-13.023	-12.018	-10.973	-9.849	-9.742	-9.857
-5	-15.026	-14.025	-13.024	-12.019	-10.978	-9.850	-9.437	-9.500
-4	-15.024	-14.024	-13.023	-12.019	-10.980	-9.870	-9.386	-9.440
-3	-15.036	-14.024	-13.023	-12.019	-10.980	-9.873	-9.379	-9.431
pH 7.4								
-9	-14.758	-13.811	-13.018	-12.393	-12.030	-12.110	-12.740	-12.924
-8	-14.751	-12.748	-12.748	-11.759	-11.107	-11.116	-11.741	-11.925
-7	-14.751	-13.750	-12.749	-11.738	-10.674	-10.179	-10.937	-10.936
-6	-14.750	-13.750	-12.750	-11.746	-10.717	-9.702	-9.873	-10.039
-5	-14.754	-13.750	-12.750	-11.747	-10.727	-9.713	-9.454	-9.539
-4	-14.749	-13.750	-12.750	-11.747	-10.728	-9.723	-9.407	-9.442
-3	-14.751	-13.750	-12.750	-11.747	-10.728	-9.724	-9.405	-9.435

References

Avdeef, A., Sofen, S.R., Bregante, T.L., and Raymond, K.N. (1978). Coordination chemistry of microbial iron transport compounds. 9. Stability constants for catechol models of enterobactin. *J. Am. Chem. Soc.* 100, 5362-5370.

King, D.W. (1998). Role of carbonate speciation on the oxidation rate of Fe (II) in aquatic systems. *Environ. Sci. Technol.* 32, 2997-3003.

Millero, F.J., and Hawke, D.J. (1992). Ionic interactions of divalent metals in natural waters. *Mar. Chem.* 40, 19-48.

Millero, F.J., Yao, W., and Aicher, J. (1995). The speciation of Fe (II) and Fe (III) in natural waters. *Mar. Chem.* 50, 21-39.

Morel, F.M., and Hering, J.G. (1993). *Principles and applications of aquatic chemistry.* New York: Wiley.

Pham, A.N., Rose, A.L., Feitz, A.J., and Waite, T.D. (2006). Kinetics of Fe(III) precipitation in aqueous solutions at pH 6.0–9.5 and 25°C. *Geochim. Cosmochim. Acta* 70, 640-650.

Pham, A.N., and Waite, T.D. (2014). Cu (II)-catalyzed oxidation of dopamine in aqueous solutions: Mechanism and kinetics. *J. Inorg. Biochem.* 137, 74-84.

Schecher, W.D., and Mcavoy, D.C. (1992). MINEQL+: a software environment for chemical equilibrium modeling. *Computers, Environment and Urban Systems* 16, 65-76.

Smith, R.M., and Martell, A.E. (1989). *Critical stability constants, Vol. 6: Second Supplement.* New York, USA: Plenum Press.

Sun, Y., Pham, A.N., and Waite, T.D. (2016). Elucidation of the interplay between Fe(II), Fe(III), and dopamine with relevance to iron solubilization and reactive oxygen species generation by catecholamines. *J. Neurochem.* 137, 955-968.
